# Effects of extracellular metabolic acidosis on the homeostasis of intracellular pH in hippocampal neurons

**DOI:** 10.3389/fphys.2024.1494956

**Published:** 2025-03-14

**Authors:** Patrice G. Bouyer, Rossana Occhipinti, Sara Taki, Fraser J. Moss, Walter F. Boron

**Affiliations:** ^1^ Department of Biology, Valparaiso University, Valparaiso, IN, United States; ^2^ Department of Physiology and Biophysics, Case Western Reserve University School of Medicine, Cleveland, OH, United States; ^3^ Auckland Bioengineering Institute, University of Auckland, Auckland, NZ, United States

**Keywords:** CO_2_/HCO_3_^−^ out-of-equilibrium solutions, pH regulation, pH_o_ sensor, HCO_3_^−^ sensor, neurons

## Abstract

This *Hypothesis* & *Theory* contribution accompanies the research paper by Bouyer et al. (Frontiers in Physiology 2024), the first to employ out-of-equilibrium (OOE) CO_2_/HCO_3_^−^ solutions to examine systematically the intracellular pH (pH_i_) effects of extracellular (o) metabolic acidosis (MAc) and its components: an isolated decrease in pH_o_ (pure acidosis, pAc) and an isolated decrease in [HCO_3_^−^]_o_ (pure metabolic/down, pMet↓). In this study, after reviewing various types of acid–base disturbances and the use of OOE solutions, we discuss pH_i_ “state” (ΔpH_i_, in response to a single acid–base challenge) and “behavior” (the ΔpH_i_ transition observed between two successive challenges), along with approaches for quantifying state and behavior. We then discuss the molecular basis of how individual extracellular acid–base disturbances influence pH_i_ via effects on—and interactions among—acid–base transporters, acid–base sensors, and cellular constitution. Next, we examine the determinants of states and behaviors, their impact on the buffering of extracellular acid loads, and how variability in state and behavior might arise. We conclude with a consideration of how mathematical models—despite their inherent limitations—might assist in the interpretation of experiments and qualitative models presented in this study. Among the themes that emerge are (1) hippocampal neurons must have distinct sensors for pH_o_ and [HCO_3_^−^]_o_; (2) these pH_o_- and [HCO_3_^−^]_o_-driven signal transduction pathways produce additive pH_i_ effects in naïve neurons (those not previously challenged by an acid–base disturbance); and (3) these pathways produce highly non-additive pH_i_ effects in neurons previously challenged by MAc.

## Introduction

Virtually, all biological processes—including those of the central nervous system—are sensitive to changes in pH. Mammals regulate the pH of the blood and extracellular fluid by adjusting the ratio of the two members of the key buffer pair: CO_2_ and HCO_3_^−^. The lungs control [CO_2_] by altering ventilation. The kidneys control [HCO_3_^−^] by altering the rate at which they secrete H^+^ into the tubule fluid and simultaneously move HCO_3_^−^ into the blood.

The acid–base status of the blood and extracellular fluid has a major influence on the pH inside the cells. Thus, we expect factors that disturb the extracellular [CO_2_]/[HCO_3_^−^] ratio to influence the acid–base status of cells, as discussed by Bouyer et al. (2024). Conversely, as cells attempt to stabilize their own pH, they move acid–base equivalents across the plasma membrane, thereby disrupting the acid–base status of the extracellular fluid.

A recent paper by Bouyer et al. (2024) explored how one particular extracellular acid–base disturbance, termed metabolic acidosis, affects the pH of rat hippocampal (HC) neurons in primary culture. As discussed in the following sections, metabolic acidosis involves a decrease in both extracellular pH and [HCO_3_^−^]. Bouyer and his colleagues used techniques to independently lower each of these parameters and, in the process, made several interesting—and in one case, startling—observations about how single and successive bouts of metabolic acidosis (or its components) affect neuronal pH_i_.

The purposes of this *Hypothesis and Theory* contribution are twofold: (1) to provide the readers of the Bouyer paper with some background for understanding the reported findings and (2) to offer potential explanations for the sometimes unexpected observations. Note that the general principles introduced in this study—for single rat HC neurons in the Bouyer paper—ought to apply to any individual eukaryotic cell, including those that are part of more complex systems such as neuron–glial co-cultures, brain slices, intact brains, diverse epithelia, and even more complex tissues like the renal cortex and blood–brain barrier. Each cell type (including different types of neurons) may require a unique set of parameters to account for their pH_i_ homeostatic mechanisms. Moreover, complex structures likely call for unique forms of cell–cell communication and, thus, control over transporters and sensors.

### Acid–base disturbances

Metabolic acidosis (MAc) is a common and potentially life-threatening acid–base disorder in mammals, including humans. It is caused by a depletion of extracellular (o) HCO_3_^−^, which leads to a decrease in both [HCO_3_^−^]_o_ and pH_o_. In a living animal, MAc generally triggers a compensatory increase in ventilation, which lowers [CO_2_]_o_ and thereby mitigates the decrease in pH_o_. Under these conditions, all three fundamental CO_2_/HCO_3_^−^ acid–base parameters underwent changes, making it difficult to attribute the effects of compensated MAc to decreased [HCO_3_^−^]_o_, decreased pH_o_, or decreased [CO_2_]_o_—or some combination of the three.

*In vitro*, we can equilibrate artificial solutions with a known partial pressure of CO_2_, thereby preventing changes in [CO_2_]_o_. Even under these conditions, however, MAc is usually associated with two altered parameters—a decrease in [HCO_3_^−^]_o_ and a decrease in pH_o_—therefore, it is still difficult to know whether the effects of MAc are due to the reduction in [HCO_3_^−^]_o_ *per se* or pH_o_ *per se*.

In addition to MAc, the three other fundamental acid–base disturbances (see Boron, 2017) are metabolic alkalosis (MAlk), in which an increase in [HCO_3_^−^]_o_ causes pH_o_ to increase; respiratory acidosis (RAc), in which an increase in [CO_2_]_o_ causes pH_o_ to decrease; and respiratory alkalosis (RAlk), in which a decrease in [CO_2_]_o_ causes pH_o_ to increase. In all of these cases, the disturbance in an intact animal leads to changes in all three acid–base parameters, along the lines discussed in the first paragraph. In the laboratory, it is possible—under equilibrium conditions—to change two at a time.

A breakthrough occurred in 1995 with the development of a rapid-mixing approach for generating out-of-equilibrium (OOE) CO_2_/HCO_3_^−^ solutions (Zhao et al., 1995), which—over a wide range of pH values—can have any combination of [CO_2_]_o_, [HCO_3_^−^]_o_, and pH_o_.

The use of OOE solutions offers a promising approacht o determining the extent to which individual acid–base parameters contribute to the physiological effects of MAc. The first such study was by Zhao et al. (2003), who found—on a background of a normal CO_2_/HCO_3_^−^ solution—that the isolated removal of basolateral (BL; i.e., blood-side) HCO_3_^−^ from isolated, perfused proximal tubules (PTs)—leaving [CO_2_]_BL_ and pH_BL_ unchanged—caused the rate of transepithelial HCO_3_^−^ reabsorption (*J*_HCO_3__), measured over ∼20 min, to increase. Thus, this challenge—the most extreme possible example of MAc but without acidosis—produced the appropriate compensatory response.

Extending the work of Zhao and her coworkers, Zhou et al. (2005) used OOE solutions in a study in which they systematically varied [CO_2_]_BL_ between 0% and 20% (leaving [HCO_3_^−^]_BL_ and pH_BL_ fixed), varied [HCO^−^_3_]_BL_ from 0 mM to 44 mM (leaving [CO_2_]_BL_ and pH_BL_ fixed), or varied pH_BL_ from 6.8 to 8.0 (leaving [CO_2_]_BL_ and [HCO^−^_3_]_BL_ fixed). Surprisingly, they found that acute^1^ changes in pH_BL_ had no effect on *J*_HCO_3__ over the ∼20-min duration of the challenges. However, starting at conditions that mimicked the composition of normal arterial blood—[CO_2_]_o_ = 5%, [HCO_3_^−^]_o_ = 22 mM; pH_o_ = 7.40—isolated changes in [CO_2_]_o_ or [HCO_3_^−^]_o_ produced the appropriate compensatory effects:

**(1) Isolated decrease in [HCO_3_^−^]_o_ ([CO_2_]_o_ and pH_o_ constant).** Bouyer et al. (2024) named this disturbance “pure metabolic/down (pMet↓).” It is the metabolic part of MAc but without acidosis. Both Zhao et al. (2003) and Zhou et al. (2005) found that pMet↓ caused *J*_HCO_3__ to increase, which would tend to compensate for MAc.
**(2) Isolated increase in [HCO_3_^−^]_o_ ([CO_2_]_o_ and pH_o_ constant).** Bouyer et al. (2024) introduced the term “pure metabolic/up (pMet↑)” in their nomenclature to describe this disturbance. It is the metabolic part of MAlk but without alkalosis. Zhou et al. (2005) found that pMet↑ caused *J*_HCO_3__ to decrease, which would tend to compensate for MAlk.
**(3) Isolated increase in [CO_2_]_o_ ([HCO_3_^−^]_o_ and pH_o_ constant).** Bouyer et al. (2024) did not propose a name for this disturbance, but we suggest “pure respiratory/up (pResp↑),” where we understand the arrow as pertaining to [CO_2_]_o_. It is the respiratory part of RAc but without acidosis. Zhou et al. (2005) found that pResp↑ caused *J*_HCO_3__ to increase, which would tend to compensate for RAc.
**(4) Isolated decrease in [CO_2_]_o_ ([HCO_3_^−^]_o_ and pH_o_ constant).** Bouyer et al. (2024) did not propose a name for this disturbance, but we suggest “pure respiratory/down (pResp↓),” where we again understand the arrow as pertaining to [CO_2_]_o_. It is the respiratory part of RAlk but without alkalosis. Both Zhao et al. (2003) and Zhou et al. (2005) found that pResp↓ caused *J*_HCO_3__ to decrease, which would tend to compensate for RAlk.

In a somewhat different protocol, Bouyer et al. (2003) started with a rabbit PT exposed on both the apical (i.e., lumen) and basolateral sides to a CO_2_/HCO_3_^−^-free solution. Adding equilibrated CO_2_/HCO_3_^−^ to the basolateral side caused a rapid increase in [Ca^2+^]_i_, whereas adding CO_2_/HCO_3_^−^ to the lumen had no effect on [Ca^2+^]_i_. Switching to an OOE basolateral solution that contained physiological CO_2_ but not HCO_3_^−^ (“pure CO_2_”) replicated the increase in [Ca^2+^]_i_, whereas switching to an OOE basolateral solution that contained physiological HCO_3_^−^ but not CO_2_ (“pure HCO_3_^−^”) had little effect on [Ca^2+^]_i_. Thus, it may be that it is basolateral CO_2_—in part acting through Ca^2+^—that triggers an increase in *J*_HCO_3__ in PTs. With our current knowledge of receptor protein tyrosine phosphatase γ (RPTPγ), we would now hypothesize that—if we started with equilibrated CO_2_/HCO_3_^−^ in the luminal and basolateral solutions—an isolated decrease in [HCO_3_^−^]_o_ would have the same effect on [Ca^2+^]_i_ as would increasing [CO_2_]_o._

The results of Zhao et al. (2003), Bouyer et al. (2003), and Zhou et al. (2005) were the first to unequivocally demonstrate that, independent of pH, each of the two components of the major blood buffer—CO_2_ and HCO_3_^−^—can act as acute, potent modulators of a biological function.

### Neuronal pH_i_ homeostasis in the face of metabolic acidosis

In an earlier study of cultured rat neurons, Bouyer and colleagues (2004) examined the effects of all four fundamental acid–base disturbances on the pH_i_ of both medullary-raphé (MR) neurons and HC neurons. For MAlk, RAc, and RAlk (but not MAc), both MR and HC neurons exhibited fully reversible pH_i_ changes, with ΔpH_i_/ΔpH_o_ ratios of ∼60%. For MAc, the responses were more intriguing. Although most MR neurons and some HC neurons exhibited a ΔpH_i_/ΔpH_o_ of ∼65%, some MR neurons and most HC neurons exhibited a ΔpH_i_/ΔpH_o_ of only ∼9% (Bouyer et al., 2004). Later, Salameh and colleagues (2014) coined the terms “MAc-sensitive” and “MAc-resistant” to describe cells like those reported by Bouyer in response to a single acid–base challenge. Interestingly, and apropos of the most recent paper by Bouyer et al. (2024), Bouyer’s 2004 neurons that we would now term MAc-resistant, when switched from a MAc solution to a control solution, they often exhibited a pH_i_ rebound to a value above the initial baseline pH_i_. A theoretical analysis led Bouyer et al. (2004) to hypothesize that the MAc-resistant neurons have a sensor for extracellular HCO_3_^−^ and that a decrease in [HCO_3_^−^]_o_ triggers an immediate stimulation of neuronal acid–base transporters that minimizes the MAc-induced decrease in pH_i_.

Salameh et al. (2014), based on observed MAc-induced pH_i_ changes in 10 cell types, proposed that the demarcation between MAc-resistant and MAc-sensitive is a (ΔpH_i_)/(ΔpH_o_) of 40%. They pointed out that any such quantitative criterion is somewhat arbitrary.

Salameh et al. (2014) also extended the protocol of Bouyer et al. (2004) by including two successive MAc challenges, MAc_1_ and MAc_2_, separated by a period of recovery in a control CO_2_/HCO_3_^−^ solution. Comparing the pH_i_ induced by MAc_2_ vs. MAc_1_, they categorized neurons as “adapting” to the MAc challenge when ΔpH_i_ during MAc_2_—(ΔpH_i_)_2/MAc_—was sufficiently smaller in magnitude than (ΔpH_i_)_1/MAc_, being “consistent” if the two ΔpH_i_ values were reasonably close and “decompensating” if the magnitude of (ΔpH_i_)_2/MAc_ was sufficiently greater than that of (ΔpH_i_)_1/MAc_.

In their recent paper, Bouyer et al. (2024) expanded upon previous work by examining substitutions of pAc or pMet↓ for MAc in HC rat neurons in primary culture. They referred to resistance and sensitivity as two relative “states” of neurons, defined for single challenges (e.g., MAc_1_ and MAc_2_). They also referred to adaptation, consistency, and decompensation, defined for the transition from the first to the second challenge, as three “behaviors.”

In her PhD dissertation, Taki (2024) examined the twin challenges of MAc and RAc in murine co-cultures of HC neurons and astrocytes. Analyzing their data along the lines of Bouyer et al. (2024), Taki et al. found that the global knockout of RPTPζ, a candidate sensor of [CO_2_]_o_ and [HCO_3_^−^]_o_ expressed mainly in the central nervous system (CNS), led to much larger acidifications than those observed in cells from WT mice.

In the following sections^2^, we provide a more formal presentation of state and behavior, along with methods for assessing them.

## Out-of-equilibrium solutions

### “The basics”

In the paper by Bouyer et al. (2024), the major contribution is the use of OOE solutions to dissect the contributions of the two components of MAc: the decreased pH_o_ *per se* and the decreased [HCO_3_^−^]_o_ *per se*. The key to understanding OOE technology is the fact that the interconversion between CO_2_ and H_2_O, on one hand, and H^+^ and HCO_3_^−^, on the other hand, involves two reactions, one of which is very slow and the other is very fast. The OOE approach separates chemical species on opposite sides of the slow reaction in the following sequence:

$${\text{CO}}_{2}+H_{2}O\;\overset{slow}{⇌}\;H_{2}{\text{CO}}_{3}\;\overset{fast}{⇌}\;H^{+}+{\text{HCO}}_{3}^{-}.$$ (1)

Although we can independently control [CO_2_]_o_, [H^+^]_o_ (i.e., pH_o_), and [HCO_3_^−^]_o_ *per se*, we have less influence over other chemical species that depend directly or indirectly on any of the entities in the preceding two-step reaction. An important example is [CO_3_^=^]_o_, which depends on both [H^+^]_o_ and [HCO_3_^−^]_o_:

$${\text{HCO}}_{3}^{-}\;⇌\;H^{+}+{\text{CO}}_{3}^{=}.$$ (2)

Moreover, the concentration of the NaCO_3_^−^ ion pair depends on both [Na^+^]_o_ and [CO_3_^=^]_o_ (as in Equation 3):

$${\text{Na}}^{+}+{\text{CO}}_{3}^{=}⇌\;{\text{NaCO}}_{3}^{-}\;,$$ (3)

${\text{CO}}_{3}^{=}$ and NaCO_3_^−^ are important for pH_i_ homeostasis because they are potential substrates of Na^+^-coupled HCO_3_^−^ transporters as our group suggested in the 1980s and 1990s (Boron and Boulpaep, 1983; Boron and Russell, 1983; Boron, 1985; Boron and Knakal, 1989; 1992). A combination of electrophysiological and mathematical modeling approaches now shows that either Na^+^ + CO_3_^=^ or NaCO_3_^−^ is the actual substrate of both the electrogenic Na/HCO_3_ cotransporter NBCe1 and the Na^+^-driven Cl-HCO_3_ exchanger NDCBE (Lee et al., 2023). Because both transporters—and closely related members of the “solute-linked carrier” 4 (SLC4) family—play important roles in pH_i_ regulation of both neurons and astrocytes, it is instructive to consider how our experimental challenges impact [CO_3_^=^]_o_.

If we assume for a moment that the second reaction in Equation 1 is infinitely slow—and if the reaction sequence in Equation 1 represents the only significant pathway between CO_2_/H_2_O and H^+^/HCO_3_^−^—then it is easy to observe how we could control [CO_2_] independently of [H^+^] and [HCO_3_^−^] and *vice versa*. The “slow” reaction in Equation 1 is slow enough that we can exploit it to make OOE solutions. The principle behind the OOE approach is to mix, with sufficient speed, two dissimilar CO_2_/HCO_3_^−^/pH solutions.

### Other reactions and considerations

In addition to the reactions shown in Equation 1, which is typically the pathway shown in textbooks (see Boron, 2017), a parallel mechanism also converts CO_2_ to HCO_3_^−^:

CO2⏟0.0012M+OH−⏟3.16×10−7M →k1=4×103 L⋅mol−1⋅s−125 Co HCO3− . (4)

The concentration values below the braces approximately correspond to a physiological partial pressure of CO_2_ (P_CO_2__) and a pH value of 7.5 at 25°C. Multiplying these concentration values by the forward rate constant yields a reaction velocity of

$$v_{{\text{CO}}_{2}+{\text{OH}}^{-}}=\left(4\times 10^{3}\;L\cdot {\text{mol}}^{-1}\cdot s^{-1}\right)\times \left(1.2\times 10^{-3}\text{ mol}\cdot L^{-1}\right)\times \left(3.16\times 10^{-7}\text{mol}\cdot L^{-1}\right)$$

$$≅1.5\times 10^{-6}M\cdot s^{-1}=1.5\times 10^{-3}\text{mM}\cdot s^{-1}\;.$$ (5)

In the case of the first reaction in Equation 1,

CO2⏟0.0012M+H2O →k1=0.036 s−125 Co H2CO3, (6)

the forward rate constant predicts a reaction rate of

$$v_{{\text{CO}}_{2}+H_{2}O}=\left(3.6\times 10^{-2}\;s^{-1}\right)\times \left(1.2\times 10^{-3}\text{ mol}\cdot L^{-1}\right)$$

$$≅4.3\times 10^{-5}M\cdot s^{-1}=4.3\times 10^{-2}\text{mM}\cdot s^{-1}\;.$$ (7)

H_2_CO_3_, the product of the “CO_2_ + H_2_O” reaction in Equation 6, would rapidly break down to form H^+^ and HCO_3_^−^. Thus, it is reasonable to compare the velocity (Equation 7) of the “CO_2_ + H_2_O″ reaction in Equation 6 with that of the “CO_2_ + OH^−^” reaction in Equation 4, which is only ∼3.5% as fast (Equation 5). This is why the OH^−^reaction in Equation 4 is generally ignored at physiological blood pH. However, the OH^−^pathway in Equation 4 is strikingly pH-sensitive because as pH increases, [OH^−^] increases exponentially. Thus, at a pH value of 9.0, the velocity of the “CO_2_ + OH^−^” reaction in Equation 4 is already 11% greater than that of the “CO_2_ + H_2_O” reaction in Equation 6. At pH 10.0, it is 11-fold faster and so on.

**FIGURE 1:**
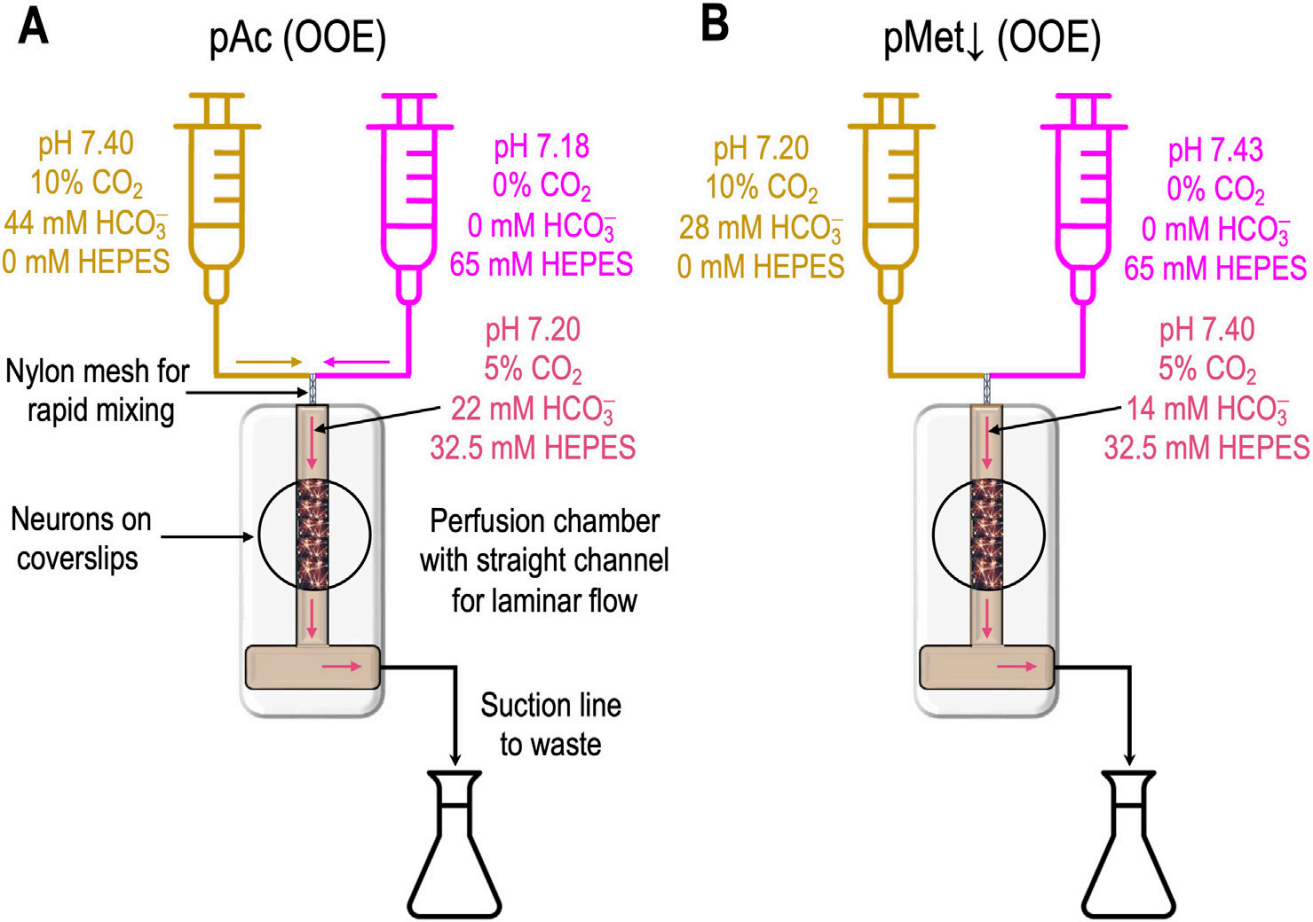
Generation of out-of-equilibrium solutions **(A)**. A heavy-duty dual syringe pump drives (at identical rates) two syringes, the acid–base contents of which are summarized by the gold-colored and magenta labels. The blended gold/magenta labels indicate the composition of the solution at the instant of mixing. The continuously generated OOE solution flows past the neurons in the chamber, before being discarded into a waste receptacle and then suctioned into an external waste container. **(B)** Generation of a pure metabolic/down (pMet↓) solution. The approach is the same as in panel A except for the contents of the two syringes.

The pH sensitivity of the OH^−^reaction has important implications for generating OOE solutions. Imagine that you wanted to generate a “pure CO_2_ solution,” one with a physiological [CO_2_] but almost no HCO_3_^−^ at pH 7.4. You might be inclined to mix a CO_2_ solution at low pH (e.g., 5.4, where most of the carbon would be in the form of CO_2_) with a CO_2_/HCO_3_^−^-free solution at a very high pH (e.g., 10). However, you will find that your final [CO_2_] will be much lower than expected, whereas your final [HCO_3_^−^] will be much higher. The reason, it seems, is that the macroscopic mixing process described in Figure 1 initially generates microdomains that contain unmixed versions of the acidic/high-CO_2_ solution and the very alkaline solution. At the interface, the reaction CO_2_ + OH^−^ → HCO_3_^−^ unexpectedly consumes your CO_2_ and disrupts your anticipated near-HCO_3_^−^-free state.

Another reaction can also wreak havoc with the creation of OOE solutions. Assume that you wanted to generate a “pure HCO_3_^−^” solution, one with a physiological [HCO_3_^−^] but almost no CO_2_ at pH 7.4. You might be inclined to mix an HCO_3_^−^ solution at a high pH value (e.g., 9.4, where most of the carbon would be in the form of HCO_3_^−^ and CO_3_^=^) with a CO_2_/HCO_3_^−^-free solution at a very low pH value (e.g., 5). However, you will find that your final [HCO_3_^−^] will be much lower than expected, whereas your final [CO_2_] will be much higher. The reason is that the hypothesized microdomains contain unmixed versions of the alkaline/high-HCO_3_^−^ solution and the very acidic solution. At the interface, the reaction H^+^ + HCO_3_^−^ → H_2_CO_3_ rapidly consumes HCO_3_^−^ while increasing [H_2_CO_3_] to very high levels, whereupon the reaction H_2_CO_3_ → CO_2_ + H_2_O disrupts your anticipated near-CO_2_-free state.

The challenges described in the preceding two paragraphs are discussed in conjunction with *figure 1* in Zhao et al. (2003).

### “Pure acidosis”

Figure 1A shows how to generate a “pure acidosis” (pAc) solution by rapidly mixing “*solution 5a*” and “*solution 5b*,” as defined in *table* ^3^ *1* in the paper by Bouyer et al. (2024). At the instant the two solutions combine, the “mixture” comprises (except for pH, which is complicated by buffer reactions) ½ A and ½ B. By trial and error (and making small pH adjustments to *solution 5b*, which contains the non-HCO_3_^−^ buffer HEPES), one can achieve the desired pH_o_ (i.e., 7.20 in the case of pAc) and the desired [CO_2_]_o_ of (10% + 0%)/2 = 5% and the target [HCO_3_^−^]_o_ of (44 mM + 0 mM)/2 + 22 mM.

The abovementioned solution is out of equilibrium at the instant of mixing but gradually degrades to equilibrium as the solution approaches the experimental chamber over a period of ∼100 ms. The [CO_2_]_o_/[HCO_3_^−^]_o_ ratio dictates a pH value of 7.4, although the actual pH_o_ value is 7.20 (i.e., higher [H^+^]_o_). Because [H^+^]_o_ is too high for the extant [CO_2_]_o_/[HCO_3_^−^]_o_ ratio, the chemical reaction as decribed in Equation 8

$$H^{+}+{\text{HCO}}_{3}^{-}\overset{fast}{\to }H_{2}{\text{CO}}_{3}\overset{slow}{\to }{\text{CO}}_{2}+H_{2}O$$ (8)

proceeds (i.e., to consume excess H^+^ so that pH_o_ will slowly increase) until both the CO_2_/HCO_3_^−^ and HEPES buffer systems are simultaneously in equilibrium. We estimate that slight (∼1%) degradation occurs as the newly mixed solution approaches the chamber and that another 1% degradation may occur as the solution flows through the chamber for removal at the other end. Thus, this technology continuously generates the desired OOE solution “online”.

### “Pure metabolic/down”

Figure 1B illustrates how to generate a “pure metabolic/down” (pMet↓)^4^ solution by mixing “*solution 6a*” and “*solution 6b*,” as defined in *table 1* in Bouyer et al. (2024). The approach is similar to that outlined above for pAc, except that our titration targets a pH_o_ value of 7.40 and a [HCO_3_^−^]_o_ value of (28 mM + 0 mM)/2 + 14 mM. In this case, the [CO_2_]_o_/[HCO_3_^−^]_o_ ratio of (5%)/(14 mM) dictates a pH value of 7.20, although the actual pH_o_ value is 7.40 (i.e., lower [H^+^]_o_). Because [H^+^]_o_ is too low for the extant [CO_2_]_o_/[HCO_3_^−^]_o_ ratio, the chemical reactions as decribed in Equation 9

$${\text{CO}}_{2}+H_{2}O\;\overset{slow}{\to }\;H_{2}{\text{CO}}_{3}\;\overset{fast}{\to }H^{+}+{\text{HCO}}_{3}^{-}$$ (9)

proceed (i.e., to generate H^+^ so that pH_o_ will slowly decrease) until both the CO_2_/HCO_3_^−^ and HEPES buffer systems are simultaneously in equilibrium.

Zhao and colleagues (2003) examined many of the technical details of employing OOE solutions, particularly in isolated, perfused renal PTs.

## State and behavior

### State

“State” describes the degree of pH_i_ change—resistant vs. sensitive—as it applies to each challenge. The state is not a quantum value—like the distinct “on” and “off” positions of a light switch—but rather like the relative brightness of a light controlled by a dimmer mechanism. The distribution of pH_i_ changes in response to MAc is more or less continuous, and the designation as resistant or sensitive is a semi-quantitative description.

**FIGURE 2:**
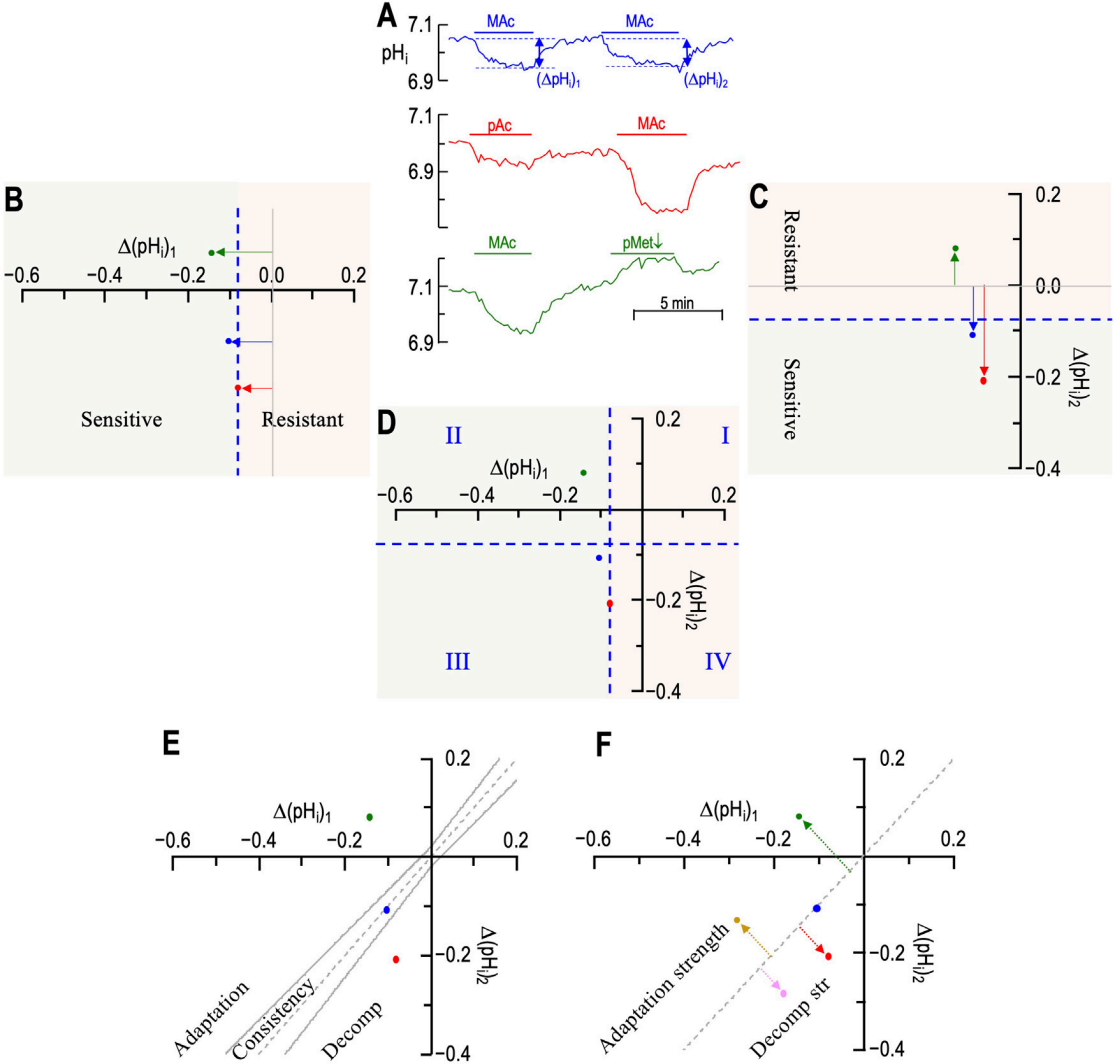
Plots of state, behavior, and behavior strength. **(A)** Three examples of experimental pH_i_ recordings. The blue record is from *figure 3a* of Bouyer et al. (2024); red, from *figure 6a* and green from *figure 9a*. **(B)** Graphical plot of “states” during the first challenge. The three horizontal arrows, with their tails on (ΔpH_i_)_1_ = 0, indicate the magnitude and direction of the pH_i_ change during challenge #1. According to the convention of Salameh et al., 2014, the vertical dashed blue line—again drawn at (ΔpH_i_)/(ΔpH_o_) = 40%—is the demarcation between the “resistant” and “sensitive” states. The green point in the positive territory indicates paradoxical alkalinization. **(C)** Graphical plot of “states” during the second challenge. The three vertical arrows, with their tails on (ΔpH_i_)_2_ = 0, indicate the magnitude and direction of the pH_i_ change during challenge #2. According to the convention of Salameh et al., 2014, the horizontal dashed blue line—drawn at (ΔpH_i_)/(ΔpH_o_) = 40%—is the demarcation between the “resistant” and “sensitive” states. **(D)** “State” diagram for twin challenges. This panel is an overlay of the previous two. I–IV indicate the quadrants formed by the two dashed blue lines. For example, the blue point in Q_III_ represents a neuron for which the state was sensitive for both challenges. **(E)** Hourglass plot for ‘behavior.’ The gray dashed line is the line of identity. For points lying on it (e.g., approximately true for blue point), (ΔpH_i_)_2_ = (ΔpH_i_)_1_. Points lying within the hourglass—formed by the upper and lower confidence limits defined by Salameh et al., 2014—define consistency of pH_i_ changes between the two challenges. Points above the hourglass represent adaptation; points below the hourglass represent decompensation (decomp). **(F)** Behavior strength (*d*_±_, *table 2* in Bouyer et al. (2024)). The arrows are orthogonal to the line of identity. Arrow length (units: pH) indicates adaptation strength (gold and green) or decompensation strength (pink and red points taken from *figure 3* of Bouyer et al., 2024).

In Figure 2A, we reproduce—for the reader’s convenience—three pH_i_ recordings from Bouyer et al. (2024). The blue record represents one of the neurons in the MAc–MAc protocol of *figure 3a* of Bouyer et al. (2024). The red record is from the pAc. MAc protocol of *figure 6a*. The green record is from the MAc-pMet↓ protocol of *figure 9a*.

In Figure 2B, the single axis (i.e., *x*-axis) represents the (ΔpH_i_)_1_ for each of the three neurons in panel A. Following the “40%” definition of Salameh et al. (2014), the vertical dashed blue line represents the demarcation between “resistant” and “sensitive” neurons for (ΔpH_i_)_1_. Because ΔpH_o_ was 0.2, this blue line is 40% × 0.2 = 0.08 pH units to the left of where the *y*-axis would be (represented by the vertical gray line). The 40% figure emanates from a study of multiple cell lines and represents a natural break in the data (see Salameh et al., 2014). Because this figure is somewhat arbitrary, one could imagine adjusting it to match the degree of MAc or the nature of a disturbance (e.g., MAc vs. MAlk vs. RAc). We have chosen to adhere to the original definition to facilitate data comparisons.

• The red point representing (ΔpH_i_)_1/pAc_ lies just to the left of the vertical dashed blue line because pAc_1_ resulted in a pH_i_ decrease of 0.084 (i.e., the point is 0.084 to the left of the vertical gray line).
• The blue point lies slightly more to the left because (ΔpH_i_)_1/MAc_ was −0.105.
• The green point lies further to the left because (ΔpH_i_)_1/MAc_ was −0.141.

All three neurons are in the green (ΔpH_i_)_1_ sensitive zone.

In Figure 2C, the single axis (i.e., *y*-axis) represents (ΔpH_i_)_2_ for each of the same three neurons in panel A. The horizontal dashed blue line represents the demarcation between “resistant” and “sensitive” neurons for (ΔpH_i_)_2_ and is 0.08 pH units below where the *x*-axis would be (represented by the horizontal gray line).

• The red point representing (ΔpH_i_)_2/MAc_ lies well below the horizontal dashed blue line because MAc_2_ resulted in a pH_i_ decrease of 0.208 (the point is 0.208 below the horizontal gray line).
• The blue point lies only slightly below the blue line because (ΔpH_i_)_2/MAc_ was −0.108.
• The green point lies paradoxically above the horizontal gray line because (ΔpH_i_)_2/pMet↓_ was +0.085.

The blue and red neurons are both in the green (ΔpH_i_)_2_ sensitive zone, whereas the green neuron is in the peach-colored (ΔpH_i_)_2_ resistant zone (which also includes paradoxical alkalinizations).

Figure 2D shows an overlay of panels B and C. The intersecting blue dashed lines now define four quadrants (Q):

• I. Any neurons in Q_I_ are resistant for both (ΔpH_i_)_1_ and (ΔpH_i_)_2_.
• II. Sensitive during (ΔpH_i_)_1_ → resistant during (ΔpH_i_)_2_.
• III. Sensitive during both (ΔpH_i_)_1_ and (ΔpH_i_)_2_
• IV. Resistant during (ΔpH_i_)_1_ → sensitive during (ΔpH_i_)_2_

### Behavior

“Behavior” describes the change in ΔpH_i_ in the transition from the first to the second challenge. By definition, behavior has meaning only for two or more challenges. We term the graphical representation of behavior the “hourglass plot” (Figure 2E), which we build around the line of identity (LoI) that describes an experimental result, in which (ΔpH_i_)_2_ = (ΔpH_i_)_1_. This is the dashed gray line running from the lower left, through the origin, to the upper right. The curved parts of the hourglass represent confidence limits, as defined by Salameh et al. (2014) and described mathematically in *equations 1* and *2* of the paper by Bouyer et al. (2024). Although the precise values of confidence limits are somewhat arbitrary, the hourglass provides an indication of the following behaviors:

• A “consistent” behavior is one in which the point representing the neuron lies within the hourglass, as typified by the blue neuron, which lies on the LoI.
• An “adaptive” behavior is one in which (ΔpH_i_)_2_ is sufficiently larger (in the algebraic sense) than (ΔpH_i_)_1_, that is, the point lies above the hourglass. The green neuron, although hardly typical, exhibits adaptation. A more typical example would fall between the *x*-axis and the upper bound of the hourglass.
• A “decompensating” behavior is one in which (ΔpH_i_)_2_ is sufficiently smaller (in the algebraic sense) than (ΔpH_i_)_1_, that is, the point lies below the hourglass, as typified by the red neuron.

Note that—as defined by Salameh et al. (2014)—a change in state does not necessarily produce an adaptive or decompensating behavior (the change in ΔpH_i_ must be sufficiently large). Conversely, the behavior can be adaptive or decompensating, although the state does not change (e.g., a point can be above or below the hourglass in Q_I_).

### Behavior strength

**FIGURE 3:**
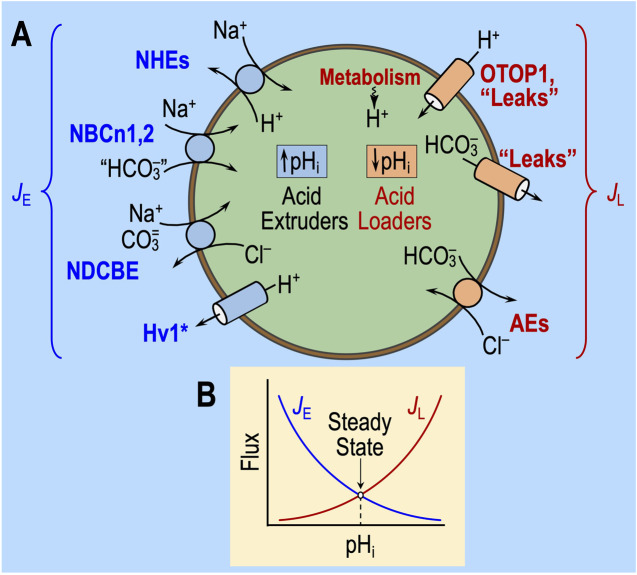
Regulation of intracellular pH. **(A)** Cell model of acid extruders (blue, on left) and acid loaders (red, on right). Acid extruders (some of which are shown in this figure) mediate the efflux of acid equivalents or the uptake of alkali equivalents. Acid loaders (some of which are shown in this figure, including cellular metabolism) mediate the uptake of acid equivalents or the efflux of alkali equivalents. Absent from this drawing are the electrogenic Na/HCO_3_ cotransporters, which seem not to make a major contribution in neurons but are extremely important in astrocytes. Some, if not all, of the Na^+^-coupled “HCO_3_^−^” transporters actually carry carbonate (CO_3_^=^) or the NaCO_3_^−^ ion pair. H^+^/monocarboxylate cotransporters are not present in this diagram. MCT1 in astrocytes mediates the efflux of lactate and H^+^ and thus operates as an acid extruder. The closely related MCT2 mediates the uptake of this lactate in neurons, where it behaves as an acid loader. *The voltage-gated proton channel Hv1 opens only at depolarized voltages and exhibits outward rectification (i.e., it operates as an acid extruder). **(B)** Kinetic model of pH_i_ regulation. The transmembrane flux is on the *y*-axis and pH_i_ on the *x*-axis. The shapes of the curves are for illustration only. *J*_E_, rate of acid extrusion from all sources; *J*_L_, rate of acid loading from all sources. When *J*_E_ = *J*_L_, pH_i_ is stable. Surface/volume ratio and buffering power have no influence on steady-state pH_i_. Cl/HCO_3_ exchanger (AE); H_V_1, voltage-gated H^+^ channel; Na-H exchangers (NHE); electroneutral Na-HCO_3_ cotransporter (NBCn); Na-driven Cl/HCO_3_ exchanger (NDCBE); other H^+^ channels (OTOP1).

The hourglass analysis provides a useful visual display. However, from a quantitative perspective, it categorizes a cell only in a ternary fashion (i.e., adaptive, consistent, and decompensating) and can categorize a population only by referring to fractions of cells with particular behaviors. Bouyer et al. (2024) introduced two variations in these concepts, in which one computes the distance of a point to the LoI. Figure 2F shows five points. Blue, red, and green represent the three neurons from panel A; the pink and gold points are two arbitrary examples from *figure 3b* of the recent Bouyer paper. The dashed line associated with each point represents the distance from the point to the LoI.

In one variation, the distance is unsigned (*d*_Absolute_)—all values are positive distances—so that average *d*_Absolute_ describes the dispersion of the points from the LoI.

In the other variation, the distance is signed *d*_±_. Positive *d*_±_ values (e.g., gold and green points)—represent points above/to the left of the LoI and thus describe the strength of adaptation. Negative values (e.g., pink and red points) represent points below/to the right of the LoI and thus describe the strength of decompensation. The blue point lies virtually on the LoI and thus has a *d*_±_ value of ∼0. The mean *d*_±_ value of a population describes the overall direction and “behavior strength”—a term coined in the dissertation by Taki (2024). An advantage of the *d*_±_ approach is that one can perform statistical tests on populations of cells (e.g., wild-type vs. knockout).

## Molecular basis of the effects of extracellular acid–base disturbances

We propose that the acute^1^ response (e.g., state and behavior/*d*_±_) of a cell to single or paired acid–base disturbances depends on a combination of three factors:

(1) near-instantaneous effects on the extracellular surface of acid–base transporters, both acid extruders (factor ‘1a’) and acid loaders (factor ‘1b’);
(2) extremely rapid effects on sensors (factor ‘2’) that detect changes in extracellular parameters and then rapidly modulate the transporters in factor ‘1’; and
(3) more slowly developing changes in cellular parameters that we will term “cellular constitution”—the collection of all ion-concentration, metabolic, and signaling properties that modulate factors ‘1’ and ‘2’ over the course of the challenge and that may persist to varying extents after the removal of the challenge. Note that the actions of factors ‘1’ and ‘2’ contribute to the constitution (factor ‘3’).

An important principle is that only factors ‘1’ and ‘2’ can influence pH_i_ over the first few seconds of a challenge. Later, gradually developing changes comprising ‘3’ can contribute not only to the pH_i_ time course during the challenge but also to the response to a subsequent challenge.

Before discussing factor ‘1’ through ‘3,’ we begin by considering the influences that cause pH_i_ to change or remain stable.

### Fundamental law of pH_i_ regulation

Figure 3A illustrates the major acid-extrusion and acid-loading mechanisms in a cell such as a CNS neuron. Two reviews consider the detailed properties of these transporters, including sensitivity to acid–base challenges (Ruffin et al., 2025; Thornell et al., 2025).

As described previously (Roos and Boron, 1981; Boron, 2004; Bevensee and Boron, 2013; Occhipinti et al., 2020; Thornell et al., 2025), the fundamental law of pH_i_ regulation is

$$\frac{d{\text{pH}}_{i}}{dt}=\frac{\rho }{\beta }\cdot \left(J_{E}-J_{L}\right).$$ (10)

Here, dpH_i_/dt is the time rate of change of pH_i_; ρ is the surface-to-volume ratio of the cell; β is total intracellular buffering power; *J*_E_ is the sum of the rates of all individual acid-extrusion processes (the rates of which are *J*_E1_, *J*_E2_, etc.), such as those on the left side of Figure 3A; and *J*_L_ is the sum of the rates of all individual acid-loading processes (the rates of which are *J*_L1_, *J*_L2_, etc.), such as those on the right side of Figure 3A.

As illustrated in Figure 3B, *J*_E_ tends to increase as pH_i_ decreases, whereas *J*_L_ tends to have the opposite pH_i_ dependence. In a steady state (i.e., when dpH_i_/dt = 0), pH_i_ is stable because *J*_E_ = *J*_L_. An acid–base challenge can initiate a change in pH_i_ (i.e., displace dpH_i_/dt from 0) only by altering *J*_E_ and/or *J*_L_, which, in turn, can occur only by producing near-instantaneous effects on transporters (factor ‘1,’ above) or sensors that rapidly regulate transporters (factor ‘2’). The subsequent time course of pH_i_ depends on evolving changes in *J*_E_ and *J*_L_, which, in turn, must reflect changes in cellular properties—for example, ΔpH_i_, Δ[HCO_3_^−^]_i_, Δ[CO_3_^=^], and other downstream parameters—that secondarily modulate the pH_i_ dependence and other kinetic properties of transporters. Thus, the evolving pH_i_ dependencies of *J*_E_ and *J*_L_ determine the new steady-state pH_i_, at which *J*_E_ and *J*_L_ come into balance during the challenge. These evolving changes could not only affect what we observe as the “state” during challenge #1, but they could also be sufficiently long-lasting to affect the “state” during challenge #2, thereby revealing themselves as “behavior.”

Note that changes in ρ or β cannot affect steady-state pH_i_ and, thus, cannot underlie a resistant/sensitive phenotype (i.e., state) or an adaptive/consistent/decompensative phenotype (i.e., behavior).^5^

### Factor ‘1’: effects on acid–base transporters

In the following analyses, the effects of acid–base challenges on transporters would be rapid-onset/rapid-offset but, as noted in the previous section, could evolve during the challenge.

#### “Acidosis” (Ac)

**FIGURE 4:**
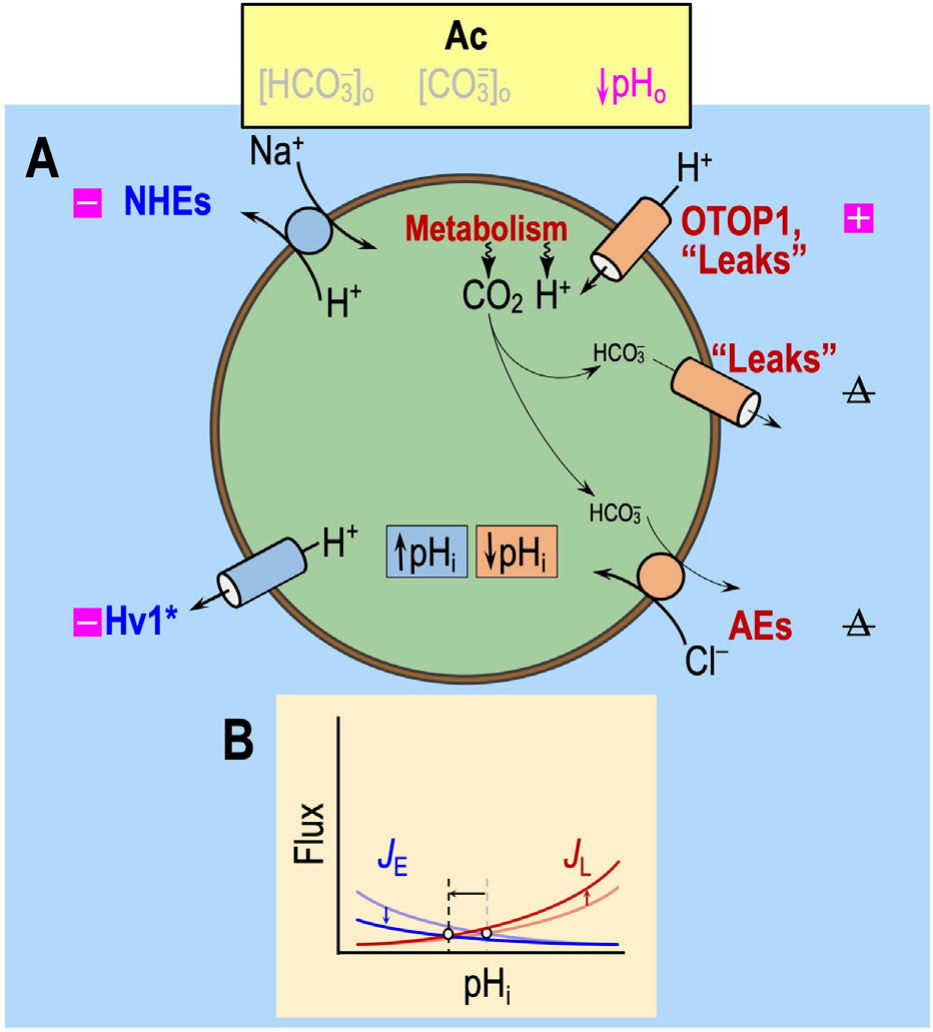
Effect of acidosis in the absence of CO_2_/HCO_3_^−^ (Ac) on transporters. **(A)** Cell model. In the nominal absence of extracellular CO_2_/HCO_3_^−^, “HCO_3_^−^” transporters have a much-reduced effect on pH_i_ homeostasis. The metabolic production of CO_2_, via the overall reaction CO_2_ + H_2_O → H^+^ + HCO_3_^−^ (likely catalyzed by carbonic anhydrases), produces HCO_3_^−^ but at levels that are most likely far lower than those observed under more physiological conditions. Thus, acid loading via HCO_3_^−^ efflux is likely to be very low. The metabolically produced CO_2_ itself exits the cell passively, either via the lipid phase of the membrane or channels (see Michenkova et al., 2021), and has no direct effect on pH_i_. Not shown in this figure—the solutions used by Bouyer et al. (2024) did not contain lactate—is the H^+^/monocarboxylate cotransporter MCT2, which physiologically mediates lactate uptake into neurons and would likely be stimulated by acidosis. **(B)** Kinetic model. In this figure, with reduced “HCO_3_^−^” transport even under control conditions, we show markedly reduced *J*_E_ (rate of acid loading from all sources) and *J*_L_ (rate of acid extrusion from all sources), as indicated by the semi-transparent blue and red curves. The more deeply colored curves indicate a *J*_E_ decrease and an *J*_L_ increase due to the effects of Ac on the pathways in panel **(A)**. The horizontal arrow represents the anticipated effect on steady-state pH_i_. Note that the removal of CO_2_/HCO_3_^−^ may lower, have no effect on, or increase steady-state pHi, depending on the initial pH_i_ and acid–base physiology of the cell. Boxes with “minus” symbols indicate inhibition, and magenta indicates a pH_o_ effect. Boxes with “plus” symbols indicate the corresponding stimulation. The struck-out Δ indicates no change. In the marquee, we indicate nominally absent parameters in gray. H_V_1, voltage-gated H^+^ channel; *J*_E_, acid-extrusion rate; *J*_L_, acid-loading rate.

In the absence of CO_2_/HCO_3_^−^, the only major acid–base transporters operative would be Na-H exchangers (NHEs) and H^+^ channels (Figure 4), as well as MCT2 monocarboxylate cotransporters, which mediate the cotransport of H^+^ and lactate. Although the physiological role of MCT2 is to import into neurons lactate generated by astrocytes (Ransom, 2017), the solutions in the paper by Bouyer et al. (2024) contain no lactate. Thus, to the extent that it operates, MCT2 would mediate H^+^/lactate efflux and—like the Na-H exchangers—function as an acid extruder. Independent of any allosteric effects, lowering pH_o_ would slow H^+^ efflux via both routes and thereby tend to lower pH_i_, as indeed Bouyer et al. (2024) observed during Ac_1_.

#### “Pure acidosis” or ↓pH_o_ (pAc)

**FIGURE 5:**
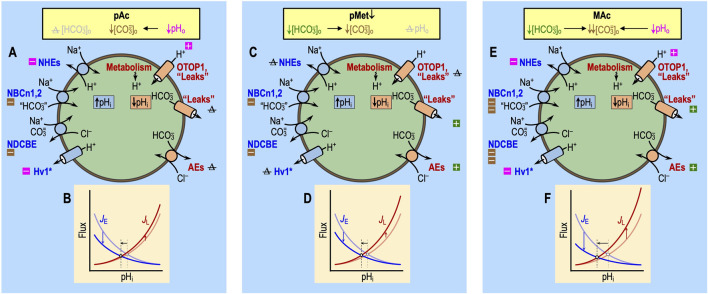
Effect of pAc, pMet↓, and MAc on transporters. Note that the only factors that can contribute to the initial (i.e., near-instantaneous) dpH_i_/dt induced by an extracellular challenge are those that immediately impact proteins facing the extracellular fluid: (1) acid–base transport pathways (including “leaks”), like those in the incomplete list shown here, and (2) rapidly responding extracellular sensors, like those shown in Figure 6. Later during the challenge, other pathways can come into play as cellular constitution changes and indirectly impacts acid–base transporters. **(A)** Pure acidosis: cellular model. The decrease in pH_o_ *per se* (magenta symbols) will produce the direct inhibition of Na-H exchangers (NHEs) and the voltage-gated H^+^ channel Hv1 and direct stimulation of other H^+^ channels like OTOP1 and “leakage” (i.e., unidentified) pathways. Indirectly, the decreased pH_o_ will lower [CO_3_^=^]_o_ (brown symbols), which will slow Na^+^-driven HCO_3_^−^ transporters—the Na^+^-driven Cl-HCO_3_ exchanger NDCBE and the electroneutral Na/HCO_3_ cotransporters NBCn1 and NBCn2—that are known or believed to carry some form of CO_3_^=^. We expect true HCO_3_^−^ pathways to be unaffected by pAc because [HCO_3_^−^]_o_ *per se* does not change. Note: pAc indicates an isolated pH_o_ decrease in the presence of CO_2_ and HCO_3_^−^ not to be confused with Ac in Figure 4, which indicates an isolated pH_o_ decrease in the absence of CO_2_ and HCO_3_^−^. **(B)** Pure acidosis: kinetic model. The semi-transparent curves—blue for *J*_E_ (rate of acid loading from all sources) and red for *J*_L_ (rate of acid extrusion from all sources)—represent control conditions and are the same as in Figure 3B. The more deeply colored curves indicate a *J*_E_ decrease and a *J*_L_ increase, both are consequences of the effects of pAc on the pathways in panel **(A)**. The horizontal black arrow represents the anticipated effect on steady-state pH_i_. **(C)** Pure metabolic/down: cell model. The decrease in [HCO_3_^−^]_o_ *per se* (green symbols) is expected to produce the direct stimulation of HCO_3_^−^ leakage pathways and Cl-HCO_3_ exchange via anion exchangers (AEs) and indirect inhibition of Na^+^-coupled HCO_3_^−^ transporters, slowing down Na^+^-driven HCO_3_^−^ transporters, which either are known or believed to carry some form of CO_3_^=^. We expect true H^+^ pathways to be unaffected by pMet↓ because pH_o_ *per se* does not change. **(D)** Pure metabolic/down: kinetic model. The meanings of the curves and symbols are the same as in panel B, compared to which we expect smaller effects on *J*_E_ but larger effects on *JL*. The horizontal arrow indicates that the decrease in pH_i_ is approximately the same length as in panel **(B)**. The data from Bouyer et al. (2024) indicate that this panel-*D* arrow should only be ∼40% as long as that in panel **(B)**. We propose that the difference could be due to the stimulatory effect of an extracellular HCO_3_^−^ sensor (see Figure 6) that would increase *J*_E_ under the conditions of pMet↓. **(E)** Metabolic acidosis: cell model. Here, we superimpose the effects of panels A and C. **(F)** Metabolic acidosis: kinetic model. Here, we superimpose the effects of panels B and D, generating a larger decrease in steady-state pH_i_ than each alone. The result of simply adding ΔpH_i_ effects in panels B and D is greater in magnitude than *Δ*pH_i_ actually observed by Bouyer et al. (2024). The reason, as suggested in the legend for panel D, may be that extracellular HCO_3_^−^ sensors (see Figure 6) reduce the decrease in pH_i_ caused by pMet↓ (see Figure 10). Boxes with “minus” symbols indicate inhibition; green indicates an effect of [HCO_3_^−^]_o_ *per se*; magenta indicates a pH_o_ effect; and brown indicates a [CO_3_^=^]_o_ effect. Boxes with “plus” symbols indicate the corresponding stimulation. The struck-out Δ value indicates no change. In the marquee, we indicate the unchanged parameters in gray. *J*_E_, acid-extrusion rate; *J*_L_, acid-loading rate.

In the presence of CO_2_/HCO_3_^−^ (Figures 5A, B), pAc would exhibit all the effects of Ac (↓ *J*_E_ and ↑ *J*_L_), presumably tending to lower pH_i_. In addition, pAc would lead to a modest decrease in [CO_3_^=^]_o_, which (because the Na^+^-coupled HCO_3_^−^ transporters appear to carry a form of CO_3_^=^; see Lee et al., 2023) would lead to a further (with respect to the one that we predict in Ac), albeit modest, decrease in *J*_E_ and, thus, a decrease in pH_i_. Finally, it is possible that the decrease in pH_o_ would have allosteric effects on various acid–base transporters, although we cannot infer the net direction without resorting to a more sophisticated quantitative approach (see Discussion).

#### “Pure metabolic” or ↓[HCO_3_^−^]_o_ (pMet↓)

Still considering events occurring in the presence of CO_2_/HCO_3_^−^, pMet↓ (Figures 5C, D) would have only one of the predicted effects of pAc: with pMet↓, the decrease in [HCO_3_^−^]_o_ would lower [CO_3_^=^]_o_ and thus modestly reduce *J*_E_. The decrease in [HCO_3_^−^]_o_ would also accelerate the efflux of HCO_3_^−^ via the Cl-HCO_3_ exchanger AE3, thereby increasing *J*_L_. Thus, the effects of pMet↓ on both *J*_L_ and *J*_E_ would tend to lower pH_i_.

#### Metabolic acidosis (MAc)

Finally, the impact of MAc (Figures 5E, F) strictly on acid–base transporters ought to be approximately the sum of the individual impacts of pAc and pMet↓, adjusted for the non-additive effects on [CO_3_^=^]_o_, as discussed by Bouyer et al. (2024).^6^

In this section, we have limited ourselves to the direct effects of challenges on acid–base transporters. In the next two sections, we will see that these are only the first part of the story: ΔpH_o_ and Δ[HCO_3_^−^]_o_ also have direct effects on sensors and indirect effects on cellular constitution, both of which are likely to modulate acid–base transporters and thus affect the pH_i_ time course. Later, we will consider the combined effects of acid–base disturbances on all three factors, namely, transporters, sensors, and constitution.^7^ Moreover, Figure 11 illustrates the apparent additivity of pAc_1_ and pMet↓_1_. In conjunction with Figure 13, we will discuss the non-additivity of pAc_2_ and pMet↓_2_.

### Factor ‘2’: effects on sensors of the extracellular acid–base status

**FIGURE 6:**
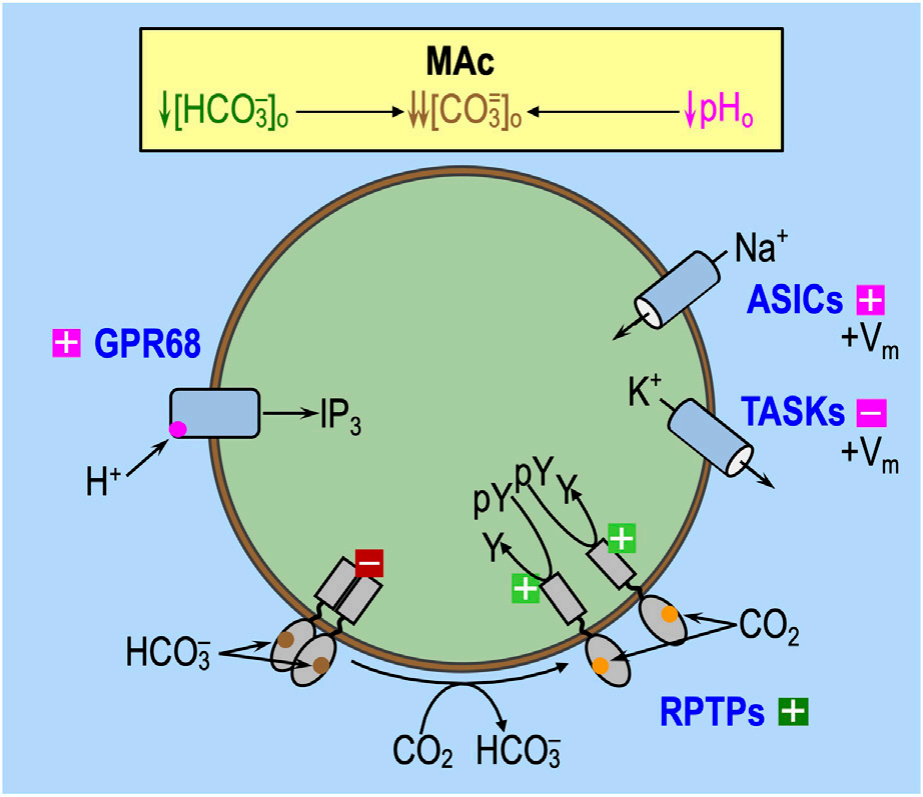
Effect of MAc on extracellular acid–base sensors. Note that the only factors that can contribute to the initial (i.e., near-instantaneous) dpH_i_/dt induced by an extracellular challenge are those that immediately impact proteins facing the extracellular fluid: (1) acid–base transport pathways (including “leaks”), like the ones in the incomplete list shown in Figure 5E, and (2) rapidly responding extracellular sensors, like the ones shown here. Later during the challenge, other pathways can come into play as cellular constitution changes and indirectly impacts acid–base transporters. GPR68 (OGR1) and at least three other G-protein-coupled receptors can sense H^+^ or a pH_o_^−^ sensitive metabolite and lead to an increase in IP_3_/Ca^2+^. The ASICs and TASKs are families of pH_o_^−^ sensitive channels. In both cases, decreases in pH_o_ lead to depolarization of the membrane, which, in turn, could have other signaling effects. In cells (e.g., astrocytes) with substantial electrogenic Na/HCO_3_ cotransporter activity, MAc would lead to decreased Na^+^ and CO_3_^=^ influx (or increased efflux), with the effect of augmenting depolarization. RPTPγ and RPTPζ have, in common, the presence of an extracellular carbonic-anhydrase–like domain (CALD), hypothesized to bind either HCO_3_^−^ or CO_2_. In the monomeric state—hypothesized to be favored by low [HCO_3_^−^]_o_—the active tyrosine phosphatase dephosphorylates tyrosine residues. Extracellular boxes with “minus” symbols indicate inhibition, and magenta indicates an effect of low pH_o_ *per se*. Extracellular boxes with “plus” symbols indicate stimulation by low pH_o_ (magenta) or low [HCO_3_^−^]_o_ (dark green). The intracellular dark-red box with a “minus” symbol indicates blockade of tyrosine phosphatase activity. The light-green box with a “plus” symbol indicates an active tyrosine phosphatase. IP_3_, inositol trisphosphate; pY, phosphotyrosine group; Y, tyrosine.

The introduction of the paper by Bouyer et al. (2025), the review by Ruffin et al. (2025), and the work by Thornell et al. (2025) summarize several classes of acid–base sensors. GPR68 (OGR1) is one of at least four pH_o_-sensitive G-protein-coupled receptors (GPCRs) and is present in medulloblastoma tissue (Huang et al., 2008), rat HC neurons (Schneider et al., 2012), and rat anterior pituitary gland (Horiguchi et al., 2014). Figure 6 depicts GPR68, in particular, and the presumed effects of MAc. HC acid-sensing ion channels (ASICs) (Alvarez et al., 2003) could play a role as pH_o_-sensors. On the other hand, although the tandem pore domain acid-sensing K^+^ (TASK) channels are present in multiple brain regions (Lesage, 2003), they are not in the hippocampus. Finally, the putative extracellular CO_2_/HCO_3_^−^ sensors, RPTPγ and RPTPζ, are widely distributed in the CNS (Müller et al., 2004; Hayashi et al., 2005; Lamprianou et al., 2006) and could potentially contribute to the pH_i_ physiology in the study by Bouyer et al. (2024). Recent work by Taki et al. (2024) shows that murine HC neurons (but not astrocytes) express both RPTPγ and RPTPζ. Moreover, in her PhD dissertation, Taki (2024) showed that the global knockout of RPTPζ in mice greatly reduces the ability of HC neurons to resist the pH_i_ decrease caused by MAc or RAc.

The activation of extracellular acid–base sensors, with a slight delay, could modulate the activity of acid–base transporters and thereby contribute to—or oppose—the initiation of pH_i_ changes predicted in Figure 5 during an acid–base challenge. The continuing actions of these extracellular sensors—that is, their effects on transporters and cellular constitution—likely impact the evolution of the pH_i_ change later during the challenge and produce longer lasting effects that influence “behavior” in the second of two challenges.

#### Effect of pAc on extracellular sensors

In the experiments of Bouyer et al. (2024), Ac (see Figure 4) and pAc (see Figures 5A, B) could act through pH-sensitive GPCRs and ion channels, which, in principle, could alter the (*J*_E_–*J*_L_) balance and thereby contribute to “state” (i.e., resistance vs. sensitivity). In Figure 6, the magenta “plus” and “minus” symbols indicate the anticipated effects of pAc on extracellular sensors.

#### Effect of pMet↓ on extracellular sensors

With pMet↓ (see Figures 5C, D), the decreased [HCO_3_^−^]_o_ would trigger HCO_3_^−^ sensors (Figure 6). In the experiments of Bouyer et al. (2024), pMet↓_1_ is unique among acid–base challenges in producing only about half the acidification of the other challenges (i.e., MAc_1_, Ac_1_, and pAc_1_). pMet↓ could promote monomerization of RPTPγ (see Figure 6), as suggested by preliminary data (Moss et al., 2018), and thereby increase the tyrosine phosphatase activity. In renal proximal tubules, it appears that this action would increase *J*_E_. Following this logic, pMet↓—acting through RPTPγ (and possibly also RPTPζ)—could promote a resistant state and, if persistent, could promote adaptation behavior in a later challenge. In Figure 6, the dark-green extracellular “plus” symbol indicates the anticipated effects of pAc on extracellular sensors.

#### Effect of MAc on extracellular sensors

The most straightforward hypothesis might be that the integrated “sensor” effects of pAc and MAc, described above (Figure 6), would summate to produce the integrated “sensor” effects of MAc (see extracellular “plus” and minus symbols in Figure 6). This may or may not be true in naïve neurons, as shown in Figure 8. However, for neurons previously exposed to MAc_1_, the integrated “sensor” effects of pAc_2_ and pMet↓_2_ may interfere with one another, as hypothesized in the discussion of Figure 14.

### Factor ‘3’: effects on cellular constitution

**FIGURE 7:**
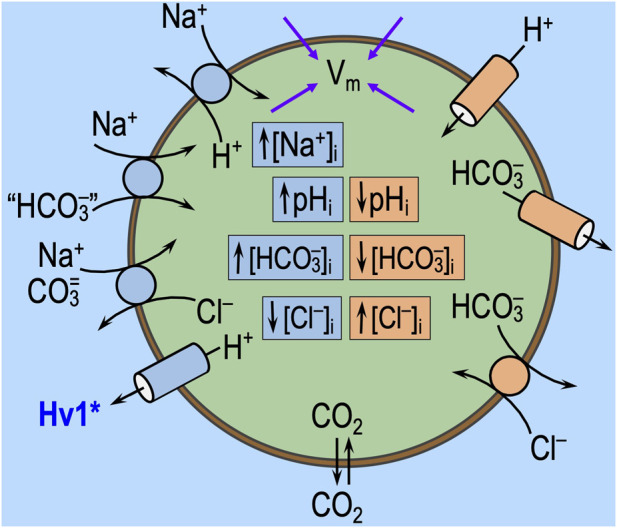
Direct effects of acid–base transport on *V*_m_ and intracellular ion concentrations. In this figure, we show the acid–base transport pathways from Figure 3, with blue and peach-colored boxes indicating the normal effects of these pathways on intracellular pH (pH_i_) and ion concentrations. We also show membrane potential (*V*_m_), which is determined by intracellular solute concentrations and the state of ion channels and electrogenic transporters. Extracellular acid–base disturbances, like those shown in Figure 5, trigger direct changes in transport activity. Extracellular acid–base sensors (see Figure 6) may modulate this transporter activity. If these transport pathways undergo net stimulation (or inhibition), the concentration changes shown in this figure will be accentuated (or attenuated). The arrows leading to *V*_m_ indicate that the rapid extracellular challenges or slower intracellular concentration changes can alter *V*_m_. In addition to the “direct” effect of changes in transport on the cellular constitution and the “secondary” effects of the extracellular sensors, we expect more complex changes to evolve over time. These complex changes could affect a myriad of membrane proteins and metabolic/signaling pathways, thereby altering the activity of the acid–base transport pathways in ways that influence “state” and “behavior.”

The effects of acid–base disturbances on transporters (see factor ‘1,’ just above) and extracellular sensors (see factor ‘2,’ above) could begin instantaneously or nearly so and continue throughout the challenge (Figure 7). Although, upon the removal of the challenge, the effects on transporters and sensors *per se* may cease just as instantaneously as they had commenced, the more slowly developing consequences of altered transporter activity on intracellular solute concentrations (e.g., pH_i_, [HCO_3_^−^]_i_, [CO_3_^=^]_i_, [Na^+^]_i_, and [Cl^−^]_i_), membrane potential (*V*_m_), and of altered sensor activation on downstream signaling pathways (e.g., phosphorylation state and protein trafficking) could evolve during the acid–base challenge and also persist for some time.^8^ In addition to constitutional changes produced directly by acid–base transporters (‘1’) and extracellular sensors (‘2’), indirect influences could include myriad effects. For example, *V*_m_ changes could affect voltage-sensitive channels and transporters and thereby affect neuronal firing and such parameters as [Ca^2+^]_i_. Alterations in ion concentrations would impact transporters and channels other than those depicted in Figure 3. For example, increased [Na^+^]_i_ would stimulate the Na–K pump, which would tend to lower [Na^+^]_i_, increase [K^+^]_i_, and hyperpolarize the cell. Changes in pH_i_ could directly impact pH_i_ sensors (reviewed by Thornell et al., 2025) and—because [HCO_3_^−^]_i_ changes in the same direction as pH_i_—could secondarily impact the soluble adenylyl cyclase sAC (Chen et al., 2000), present in some HC axon terminals (Chen et al., 2013). In locus coeruleus chemosensitive neurons, the activation of sAC increases L-type Ca^2+^-currents and limits the hypercapnia-induced increase in the firing rate (Imber et al., 2014).

The above mentioned effects could produce changes in the number of acid–base transporter proteins in the plasma membrane (due to trafficking, protein degradation, and eventually protein synthesis) and changes in their unitary or “per-molecule” activities (due to alterations in intracellular ionic and post-translational modifications). Thus, the “functional activity” of transporters (i.e., protein number × unitary activity) underlying many *J*_E1_, *J*_E2_, … and *J*_L1_, *J*_L2_, … terms introduced in our introduction of Equation 10 may change over the evolution of the acid–base disturbance and then persist for some time.

Taki (2024) suggests that in a MAc–MAc protocol, progressively lower and lower pre-MAc_2_ pH_i_ values correlate with an increase in the degree of adaptation behavior. Because higher pH_i_ values just before MAc_2_ translate to higher [HCO^−^_3_]_i_ values just before MAc_2_ (assuming that CO_2_ has equilibrated across the cell membrane), it is possible that sAC (which senses cytoplasmic HCO_3_^−^) could participate in neuronal state and/or behavior. Other pH_i_-sensitive processes could respond during a challenge, and the extracellular sensors could affect these or *vice versa*.

In acutely dissociated HC CA1 neurons, Brett et al. (2002) have shown that the inhibition of protein kinase A (PKA) inhibits Cl-HCO_3_ exchange but stimulates Na^+^-dependent Cl-HCO_3_ exchange, thereby increasing pH_i_ in low-pH_i_ neurons. In high-pH_i_ neurons, the effects are the opposite. The stimulation of PKA has the opposite set of effects. In the protocols of Bouyer et al. (2024), decreases in pH_o_ could have activated pH_o_^−^ sensitive GPCRs that elevate [cAMP]_i_ (Radu et al., 2005) and thereby contributed to state and behavior.

## Determinants of neuronal state and behavior

Even before the work of Bouyer et al. (2024), Bouyer et al. (2004) had shown that some HC and medullary raphé neurons exhibit smaller pH_i_ decreases than other neurons—what Salameh et al. (2014) would later term MAc resistance vs. sensitivity. Salameh et al. (2014) later showed that resistance/sensitivity and adaptation, consistency and decompensation phenotypes occur in multiple cell types other than HC neurons and astrocytes.

We hypothesize that state—resistance vs. sensitivity—depends both on the pre-existing status of the three factors discussed above and how constitutional changes evolve during the challenge. The pre-existing status, which could reflect the previous history of acid–base and other challenges, comprises the kinetic properties of each acid–base transporter and all factors (e.g., the impact of extra- and intracellular sensors) that influence these kinetic parameters.

We hypothesize that behavior—adaptation vs. consistency vs. decompensation—depends on all of the elements that determine the state during the first of two challenges and the persistence of all changes in cellular parameters from the first challenge to the next. Presumably, these parameter changes eventually extinguish with time. However, to the extent that the changes persist, they represent a sort of memory of the previous challenge that influences how a cell responds to a future challenge. Examples of persistent changes could include alterations in the numbers of various acid–base transporters and sensors that are resident in the plasma membrane, their post-translational states, and cellular constitution.

Although it was outside the scope of the study by Bouyer et al. (2024), it would be illuminating to examine the challenges opposite to those in that study (i.e., metabolic alkalosis or MAlk, pure alkalosis or pAlk, and pure metabolic/upward or pMet↑), as well as respiratory acidosis (RAc) and alkalosis (RAlk), pure respiratory/up (an isolated increase in [CO_2_]_o_ or pR↑), and pure respiratory/down (pR↓). Note, however, that in the study by Bouyer et al. (2004), it was MAc—not RAc, MAlk, or RAlk—that seemed to generate pH_i_ responses that were the most idiosyncratic.

### State: resistance vs. sensitivity

**FIGURE 8 F8:**
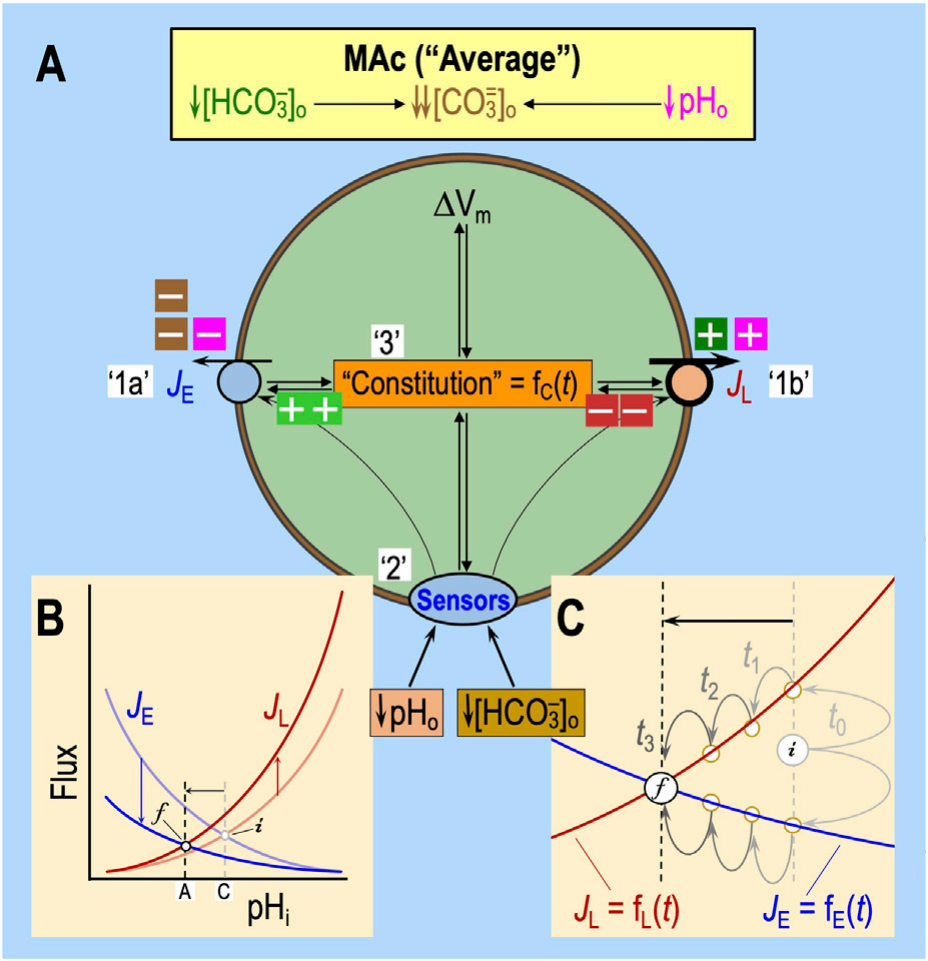
Model of average “state” during MAc. **(A)** Cellular model of the effects of MAc. In this figure, we suppose a state response to MAc that is on the border of resistant and sensitive—that is, “average.” The blue *J*_E_ symbol represents the total flux mediated by all mechanisms of acid extrusion (factor ‘1a’), whereas the red *J*_L_ symbol includes fluxes of all mechanisms of acid loading (factor ‘1b’ in white box). The oval “Sensors” symbol includes all sensors that respond to changes in [HCO_3_^−^]_o_ or pH_o_ (factor ‘2’). RPTPγ and RPTPζ presumably also respond to changes in [CO_2_]_o_, which did not occur in the experiments conducted by Bouyer et al. (2024). The extracellular dark-green, brown, and magenta “plus” and “minus” symbols have the same meaning as detailed in the previous figures (i.e., indicating which aspect of the MAc challenge produces the stimulation or inhibition, as shown in Figure 5E). The intracellular light-green “plus” and dark-red “minus” symbols (emanating from “Sensors” and Constitution) indicate enhancement or depression. Although we show equal numbers of intracellular light-green “plus” symbols and red “minus” symbols, it is really some combination of the two that reflects the relative degrees of transporter stimulation/inhibition by “Sensors” and/or “Constitution.” The black double arrows indicate that *J*_E_ influences cellular constitution (factor ‘3’) and *vice versa*. The same holds true for Δ*V*_m_, *J*_L_, and the hypothesized sensors (see Figure 6) for extracellular H^+^ (e.g., GPR68) and HCO_3_^−^ (e.g., RPTPγ and ζ). Note that we hypothesize that constitution is a function of time. **(B)** Kinetic model. This panel is a reproduction of the material shown in Figure 5F. **(C)** Higher magnification view of the kinetic model shown in panel **(B)**. As illustrated in panel B, MAc instantly causes the *J*_E_ curve to shift downward and the *J*_L_ curve to shift upward, as indicated by the more deeply colored blue and red curves, respectively. In this figure, in panel C, we reproduce, at higher magnification, the newly shifted *J*_E_ (blue) and *J*_L_ (red) curves, the two vertical dashed lines, the horizontal arrow, and the points that we label “i” (initial) and “f” (final). Before MAc, the semi-transparent blue and red curves (shown in panel B but not C) passed through point “i.” Upon the imposition of MAc, at time “t_0_,” the *J*_E_ value instantaneously jumps upward to meet the more deeply colored red curve, as indicated by the upper light gray arrow, and the *J*_L_ value instantly jumps downward to meet the more deeply colored blue curve. Because *J*_L_ > *J*_E_, that is, Δ(*JE–JL*) is negative, pH_i_ begins to decrease at its maximal rate for this experiment. As pH_i_ decreases (moving leftward on red and blue curves), *J*_L_ decreases and *J*_E_ increases. After time *t*_1_, Δ(*JE–JL*) is still negative but to lesser extent than at time *t*_0_. Thus, pH_i_ decreases more slowly, eventually reaching time *t*_3_, where *J*_E_ and *J*_L_ come back into balance—that is, Δ(*JE–JL*) = 0—so that pH_i_ is in a new, lower steady state at point “f” than during control conditions at point “i.” Because cellular constitution changes during the MAc challenge, *J*_E_ and *J*_L_ are both functions of time.

Salameh et al. (2014) defined MAc-resistant cells as those for which pH_i_ decreases by <40% of ΔpH_o_. Regardless of where one draws the dashed blue lines in Figures 2B, D, some cells will be more resistant/sensitive than others. Bouyer et al. (2024) observed a continuum of ΔpH_i_ values that presumably depend on the factors noted in the previous section:^9^ rapid effects on ‘1’ acid–base transporters, ‘2’ extracellular acid–base sensors, and ‘3’ more slowly developing effects on cellular constitution. Figure 8A summarizes the interdependence of factors ‘1’–‘3’ for a naïve cell with an “average^10^” pH_i_ decrease during MAc_1_. The initial (*i*) steady-state pH_i_ (i.e., pH_i_ prevailing just before MAc) is described by the intersections of the semi-transparent blue and red curves. We now discuss the impact of MAc_1_ on cells in four different states—sensitive and resistant plus “average” and “paradoxical” (an extreme variant of resistant)—and then raise the issue of how pAc_1_ and pMet↓_1_ contribute to MAc_1_.

#### “Average” cells

Viewed in the context of Equation 10, for all but a small fraction of cells with paradoxical responses (discussed below^11^), the imposition of MAc temporarily shifts the difference (*J*_E_–*J*_L_) in the negative direction (see Figure 8B), initiating a decrease in pH_i_ that plays out over several minutes. At the instant of the switch to MAc (see Figure 8C, *t*_0_), pH_i_ has not yet changed. Nevertheless, *J*_E_ jumps to the new *J*_E_ vs. pH_i_ curve (bright blue), which we presume to be below the original one. Simultaneously, *J*_L_ jumps to the new *J*_L_ vs. pH_i_ curve, which we presume to be above the original one.^12^ As a result, *J*_L_ exceeds *J*_E_ at *t*_0_, and pH_i_ begins to decrease at a rate determined by ρ, β, and Δ(*J*_E_–*J*_L_) in Equation 10. As pH_i_ declines, *J*_E_ increases gradually (*t*_1_, *t*_2_, and *t*_3_) and *J*_L_ decreases. At *t*_3_, *J*_E_ and *J*_L_ have once more attained a balance at the final (*f*) steady state. Although Figure 8C depicts the *J*_E_ and *J*_L_ curves as being static (i.e., having fixed shapes and positions in the two-dimensional space of the chart), the shapes and positions of *J*_E_ vs. pH_i_ and *J*_L_ vs. pH_i_ could evolve over time, in response to changes in the extracellular sensors and cellular constitution, both of which potentially impact *J*_E_ and *J*_L_.

#### Sensitive cells

**FIGURE 9 F9:**
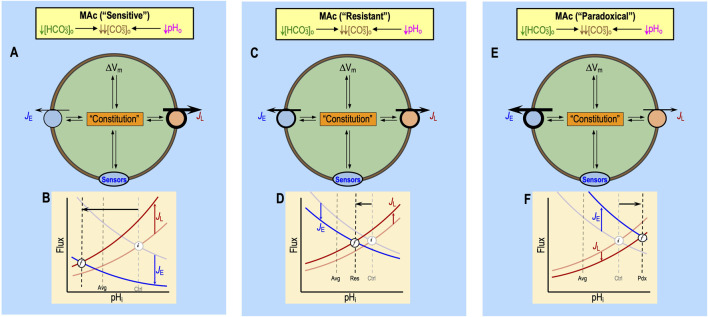
Models of sensitive, resistant, and paradoxical states during MAc. We hypothesize that MAc produces the usual initial percent inhibition (extracellular “minus” symbols) or stimulation (extracellular “plus” symbols) of each transporter (see Figure 5E) and sensor (see Figure 6), regardless of the subsequent pH_i_ response that is indicative of state—sensitive, resistant, or paradoxical (exaggerated version of resistant). Instead, differences in the state would reflect differences in (1) transporter numbers, (2) sensor numbers, and (3) cellular constitution (which would influence the intrinsic transporter and sensor activity). In the cellular model panels **(A, C, E)**, the thicknesses of arrows for *J*_E_ (rate of acid loading from all sources) and *J*_L_ (rate of acid extrusion from all sources) reflect functional activities (i.e., product of the protein number and intrinsic activity per protein). In the kinetic model panels **(B, D, F)**, the semi-transparent curves (blue for *J*_E_ and red for *J*_L_) are the same as the curves shown in Figure 3B; their intersections reflect initial (*i*) pH_i_ values. The more deeply colored curves indicate the hypothetical *J*_E_ and *J*_L_ curves that prevail in each of the three states, and their intersections reflect final **(F)** pH_i_ values. The horizontal black arrows represent the anticipated effect on steady-state pH_i_ (i.e., *i* → **(F)**. **(A)** MAc-sensitive neuron: cellular model. The sensitive state, reflecting the status of sensors and cellular constitution, results in some combination of depressed acid extrusion and elevated acid loading. **(B)** MAc-sensitive neuron: kinetic model. The deeply colored curves indicate a large *J*_E_ decrease and a large *J*_L_ increase due to the effects of MAc on the pathways in panel A for this neuron with a sensitive state. The result is a large decrease in steady-state pH_i_. Note: these two curves are the most exaggerated *J*_E_ and *J*_L_ curves, compared to the “average” cell shown in Figure 8, “resistant” cell shown in Figure 9C, and “paradoxical” cell shown in Figure 9E. **(C)** MAc-resistant neuron: cellular model. The resistant state, reflecting the status of sensors and cellular constitution, results in some combination of a modest *J*_E_ decrease and a modest *J*_L_ decrease, both of which are in opposite directions compared to the “sensitive” neuron shown in Figure 9A, “average” shown in Figure 8, and “paradoxical” shown in Figure 9E. **(D)** MAc-resistant neuron: kinetic model. The deeply colored curves indicate only a modest *J*_E_ decrease vs. the larger one in panel **(B)** and a modest *J*_L_ increase vs. the larger one in panel **(B)** due to the effects of MAc on the pathways in panel C for this neuron with a resistant state. The result is only a modest decrease in steady-state pH_i_. **(E)** paradoxical response to MAc: cellular model. The paradoxical response is an extreme variant of the resistant state and reflects that the status of sensors and cellular constitution results in some combination of a robust increase in *J*_E_ and a modest decrease in *J*_L_. Note that the directions of these changes are opposite those of the “sensitive” neuron shown in Figure 9A, the “average” neuron shown in Figure 8, and the “resistant” neuron shown in Figure 9C. We propose a possible cellular mechanism of the paradoxical responses to MAc_1_ shown in Figure 10. **(F)** Paradoxical response to MAc: kinetic model. The deeply colored curves indicate some combination of a robust *J*_E_ increase (vs. the decreases in the other examples) and a modest *J*_L_ decrease (vs. the increases in the other panels) due to the effects of MAc on the pathways in panel E for this paradoxical neuron. The result is an increase in steady-state pH_i_.

For cells that respond to MAc with a relatively large pH_i_ decrease, the net effect of MAc on factors ‘1’–‘3’ must be to produce a highly negative Δ(*J*_E_–*J*_L_) over the period of the MAc challenge. Some neurons are unusually sensitive to MAc. For example, examination of *figures 3b*, *5b*, *7b*, and *9b* in Bouyer et al. (2024) reveals that, during MAc_1_, some HC neurons (a total of 35 out of 230 or ∼15.2%) exhibit a decrease in pH_i_ that is even greater in magnitude than the decrease in pH_o_ during MAc; in other words, (ΔpH_i_)_1/MAc_ < −0.20. In these neurons, MAc_1_ must have produced a sufficiently large negative shift in Δ(*J*_E_–*J*_L_), integrated over the period of the challenge, to produce an unusually large intracellular acidification. In the cell model of Figure 9A, we imagine that MAc causes a large decrease in *J*_E_ and a large increase in *J*_L_. In Figure 9B, we imagine a large downward shift (or a shallower slope) in the *J*_E_ curve and a large upward shift (or steeper slope) in the *J*_L_ relationship. Either a sufficiently large *J*_E_ downshift or *J*_L_ upshift could produce a highly negative Δ(*J*_E_–*J*_L_) and thus a highly MAc-sensitive state.

#### Resistant cells

For cells that respond to MAc with a relatively small pH_i_ decrease, the net effect of MAc on factors ‘1’–‘3’ must be to produce a negative Δ(*J*_E_–*J*_L_) that is relatively small in magnitude. In the cell model of Figure 9C, we assume that MAc causes a modest decrease in *J*_E_ and a modest increase in *J*_L_. Figure 9D represents these *J*_E_/*J*_L_ changes as a more modest *J*_E_ downshift and *J*_L_ upshift, although either effect could dominate to produce a modestly negative Δ(*J*_E_–*J*_L_) and thus a highly MAc-resistant state.

Salameh et al. (2017) revealed an interesting mechanism by which HC neurons mitigate the decrease in pH_i_ during MAc, a process that depends on changes in cellular composition. In HC neurons cultured from WT mice, MAc tends to induce a pH_i_ decrease that is initially rapid but limited in magnitude (Salameh et al., 2017). However, in HC neurons cultured from mice genetically deficient in the Cl-HCO_3_ exchanger AE3 (an acid loader), MAc induces a relatively slow initial decrease in pH_i_ (reflecting the absence of AE3 and thus a smaller, initial MAc-induced negative shift in *J*_L_) that continues for some time. The result is a slow but large decrease in pH_i_. Salameh *et al* argued that, in WT neurons, the robust activity of AE3 loads the cell with Cl^−^, which, in turn, increases *J*_E_ by stimulating both the Na^+^-driven Cl-HCO_3_ exchanger and NHEs, which often have a positive dependence on [Cl^−^]_i_ (see Parker, 1983; Davis et al., 1994; Rajendran et al., 1995, 1999; Hogan et al., 1997; Bevensee et al., 1999). We interpret this hypothesized increase in [Cl^−^]_i_ as a gradual change in cellular constitution that progressively increases *J*_E_ over time and thereby tends to bring *J*_E_ and *J*_L_ into balance at a relatively high pH_i_—that is, the WT neurons appear to be relatively resistant to MAc. Thus, we would expect that neurons with relatively high functional activities of AE3, NDCBE, or NHE would tend to be more MAc-resistant, whereas neurons with lower functional activities would tend to be more MAc-sensitive.

#### Paradoxical responses

Returning to the paper by Bouyer et al. (2024), an examination of their *figures 3b*, *5b*, *7b*, and *9b*—all of which have MAc as challenge #1—reveals that, during MAc_1_, a small fraction of HC neurons (a total of 22 out of 230 or ∼9.6%) exhibit a paradoxical alkalinization. In other words, for these 22 neurons, (ΔpH_i_)_1/MAc_ > 0, so the points representing each lie to the right of the *y*-axis in a state diagram like that in Figure 2D. The net effect of MAc_1_ in these 22 paradoxical neurons must have been to produce an immediate and sustained positive shift in Δ(*J*_E_–*J*_L_), as illustrated in Figures 9E, F.

Analogous to the 22 paradoxical pH_i_ increases discussed above is a non-physiological case that results from exposing naïve neurons to pMet↓. As summarized in *figure 8b* of Bouyer et al. (2024), 20 of 52 neurons (38%) alkalinized in response to pMet↓_1_.

We are unaware of any mechanism through which MAc_1_ (see Figures 5E, F) or pMet↓_1_ (see Figures 5C, D) could act directly on transporters to produce such an immediately positive, paradoxical pH_i_ increase. Rather, it is more likely that, in a subset of neurons, extracellular sensors detect the decrease in [HCO_3_^−^]_o_ (in MAc_1_ or pMet↓_1_) and/or pH_o_ (in MAc_1_) and respond by producing a marked and extremely rapid increase in (*J*_E_–*J*_L_) that overwhelms the more typical acidifying effects of MAc_1_ (Figures 8, 9A–F) and pMet↓_1_.

**FIGURE 10:**
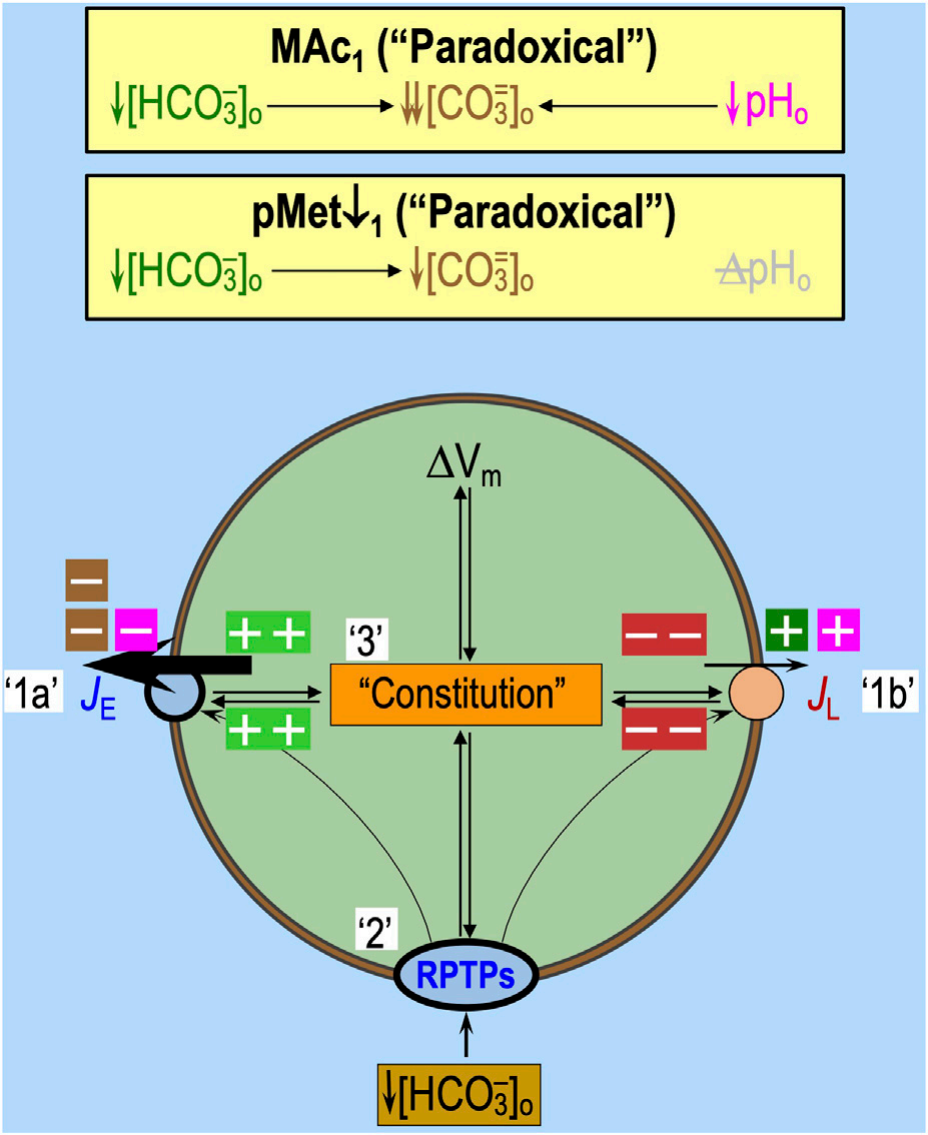
Hypothesized mechanistic model, for a naïve HC neuron, of paradoxical pH_i_ increases induced by MAc or pMet↓. We hypothesize that MAc_1_ produces the usual initial percent inhibition (extracellular brown or magenta “minus” symbols) or stimulation (extracellular dark-green or magenta “plus” symbols) of each transporter (see Figure 5E) and sensor (see Figure 6), regardless of the subsequent pH_i_ response indicative of state. pMet↓_1_ would produce only the effects indicated by extracellular dark-green and brown “minus” and “plus” symbols (i.e., not magenta symbols). Compared to other naïve neurons, the paradoxical responses to MAc_1_ or pMet↓_1_ (state) would reflect differences in (1) transporter numbers, (2) sensor numbers, and (3) cellular constitution (which would influence intrinsic transporter and sensor activity). The thicknesses of the arrows for *J*_E_ (rate of acid loading from all sources) and *J*_L_ (rate of acid extrusion from all sources) and the RPTPs (oval) reflect functional activities (i.e., product of the protein number and intrinsic activity per protein). The intracellular light-green “plus” symbols and red “minus” symbols indicate the relative effects of cellular constitution (including signaling from RPTPs) on *J*_E_ and *J*_L_. Although we show equal numbers of intracellular light-green “plus” symbols and red “minus” symbols, it is really some combination of the two that reflects the relative degrees of transporter stimulation/inhibition by “Sensors” and/or “Constitution.” We predict that pMet↓_1_, lacking the pH_o_ effects of MAc_1_, would have relatively more light-green “plus” symbols and fewer red “minus” symbols, thus indicating a greater net increase in Δ(*J*_E_–*J*_L_) and a greater paradoxical pH_i_ increase than MAc_1_.

Given that (1) an isolated decrease in basolateral [HCO_3_^−^]_o_ (delivered via an OOE solution) acutely increases *J*_E_ in renal PTs (Zhou et al., 2005), (2) PTs are insensitive to acute, isolated decreases in basolateral pH_o_ (OOE solution) during this time frame (Zhou et al., 2005), (3) the PT response requires RPTPγ (Zhou et al., 2016), and (4) RPTPγ and RPTPζ are present in virtually every mouse HC neuron (Taki et al., 2024), we propose the following mechanism (Figure 10) by which the ∼10% of naïve HC neurons subjected to MAc_1_ and the nearly 40% subjected to pMet↓ exhibit a paradoxical pH_i_ increase: the decrease in [HCO_3_^−^]_o_ triggers the monomerization of RPTPγ or RPTPζ (see Figure 6), leading to the dephosphorylation of certain phosphotyrosines and, as a consequence, the rapid stimulation of acid extruders and/or inhibition of acid loaders.

#### Additivity of pAc_1_ and pMet↓_1_

**FIGURE 11:**
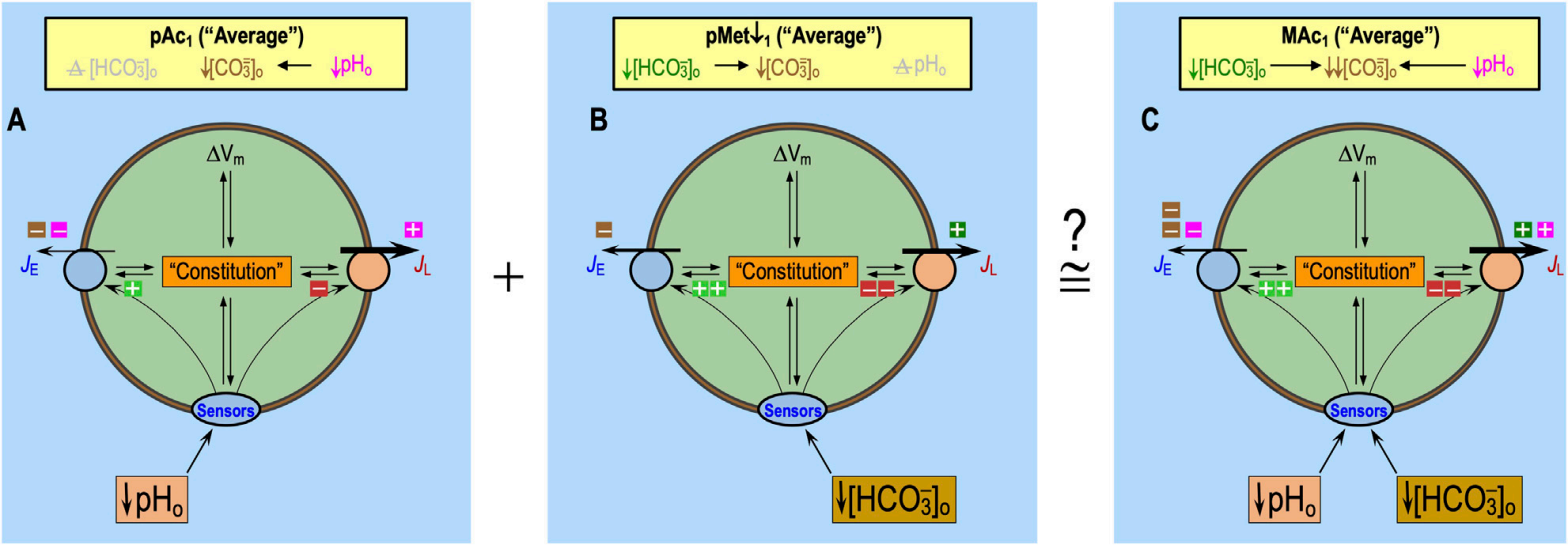
Hypothesized cellular mechanism, for naïve neurons, of additivity: pAc_1_ + pMet↓_1_ ≅ MAc_1_. **(A)** Effect of pure acidosis on an “average” HC neuron. We hypothesize that the decrease in pH_o_ stimulates only one class of sensors (e.g., GPR68) with relatively weak functional activity. **(B)** Effect of pure metabolic/down on an “average” HC neuron. We hypothesize that the decrease in [HCO_3_^−^]_o_ stimulates only one class of sensors (e.g., RPTPγ/RPTPζ) with relatively strong functional activity. **(C)** Effect of metabolic acidosis on an “average” HC neuron. We hypothesize that the simultaneous decreases in both pH_o_ and [HCO_3_^−^]_o_ stimulate both classes of sensors. The symbols have the same meanings as in previous figures. The thickness of the arrows representing *J*_E_ (rate of acid loading from all sources) and *J*_L_ (rate of acid extrusion from all sources) and the thickness of the lines surrounding “Sensors” reflect the relative functional activities. The numbers of intracellular light-green “plus” symbols and red “minus” symbols (some of which are shown as halves) reflect the degree of stimulation or inhibition by the Sensors and/or Constitution. Although we show equal numbers of intracellular light-green “plus” symbols and red “minus” symbols, it is really some combination of the two that reflects the relative degrees of transporter stimulation/inhibition by “Sensors” and/or “Constitution.”

The data from Bouyer et al. (2024) show that, in naïve neurons, the average ΔpH_i_ elicited by pAc_1_ and the average ΔpH_i_ elicited by pMet↓_1_ approximately summate to the average ΔpH_i_ elicited by MAc_1_ in a population of rat HC neurons. The reported contributions were ∼70% for pAc_1_ and ∼30% for pMet↓. Figure 11 illustrates how this additivity could occur in a single “average” neuron. Considering only the direct effects of acid–base disturbances on transporters—that is, without the effect of the hypothesized extracellular H^+^ and HCO_3_^−^ sensors—we predicted that the ΔpH_i_ effect of pMet↓_1_ would have been similar to that of pAc_1_, so the two would have summed to a ΔpH_i_ value greater than that produced by MAc_1_. Thus, we hypothesize that in naïve HC neurons, the effect of decreased pH_o_ on extracellular-H^+^ sensors produces a relatively weak stimulation of acid extrusion overloading (i.e., weak opposition to the pH_i_ decrease), whereas the effect of decreased [HCO_3_^−^]_o_ produces a relatively strong stimulation (i.e., strong opposition to the pH_i_ decrease).

#### Summary

At the population level, the “state” revealed by MAc_1_ in naïve neurons seems to be the sum of the effects of pAc_1_ and pMet↓_1_. The degree of resistance (or sensitivity) to MAc depends on how, integrated over the period of the challenge, MAc affects the (*J*_E_–*J*_L_) balance (see Equation 10). In turn, this balance depends on the cell’s complement of acid–base transporters and extracellular acid–base sensors, initial cellular constitution, and how the cell modulates these factors over the course of the MAc challenge.

### Behavior: adaptation vs. consistency vs. decompensation

The three types of behavior must reflect persistent effects (or lack thereof) on the three factors introduced above^13^ to produce, during MAc_2_, a state that is the same, more resistant, or more sensitive than during the preceding MAc_1_.

In the next three subsections, we (1) present hypotheses of how behaviors arise, (2) explore insights from the non-additivity of pAc_2_ and pMet↓_2_ [a conclusion reached in *equations 6* & *7* of Bouyer et al. (2024)], and (3) consider parameters that could affect behavior.

### Models of behaviors

**FIGURE 12:**
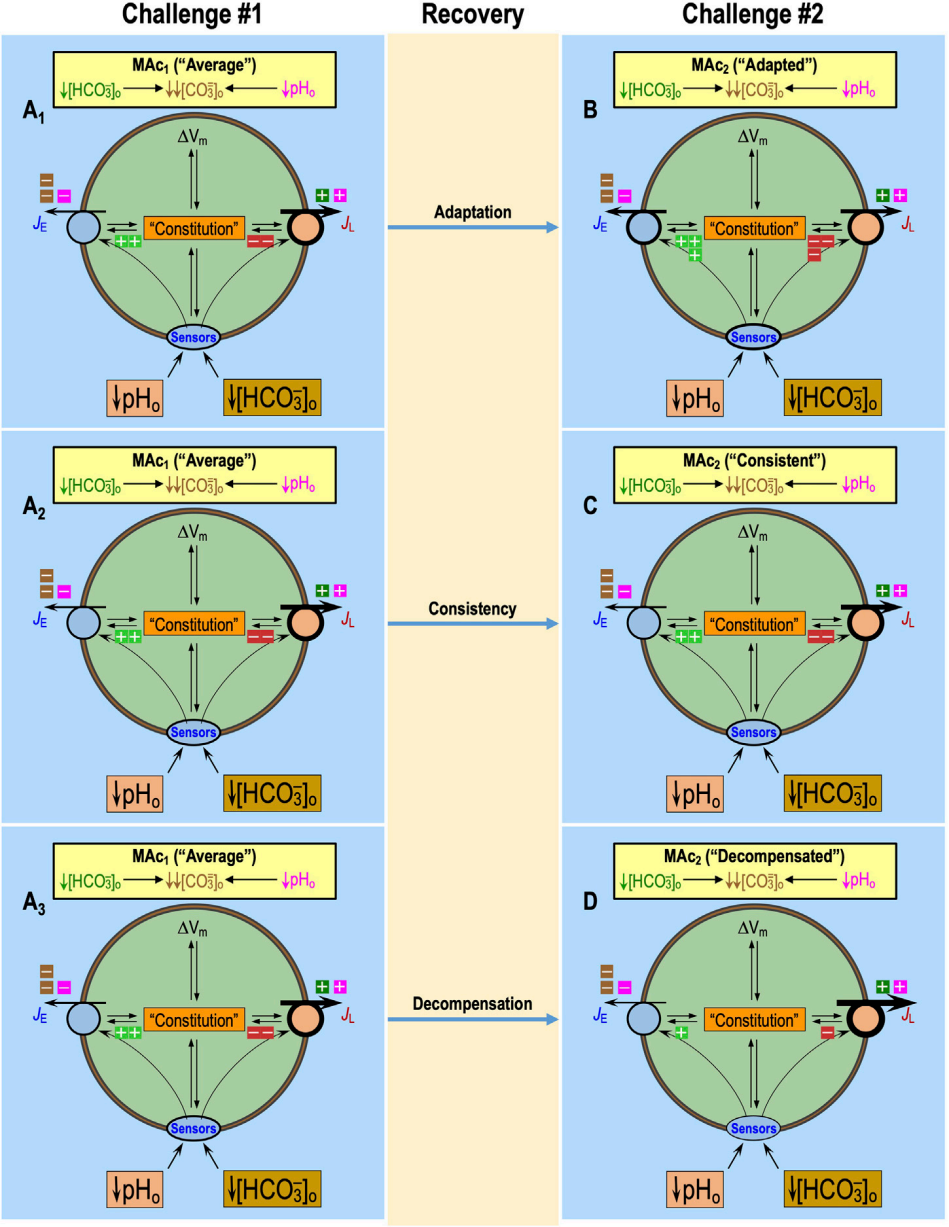
Model of the mechanisms of behavior. **(A**_**1**_ **–A**_**3**_ **)** Cell state during challenge #1. **(A**_**1**_ **–A**_**3**_ **)** These are identical and reflect the state of an “average” naïve HC neuron during the first challenge with MAc. After this MAc_1_ challenge, the neuron spends several minutes in a recovery period, exposed to a control CO_2_/HCO_3_^−^ solution. The thicknesses of arrows for *J*_E_ (rate of acid loading from all sources) and *J*_L_ (rate of acid extrusion from all sources) reflect functional activities (i.e., product of the protein number and intrinsic activity per protein). **(B)** State of an adapted neuron during a second MAc challenge. We hypothesize that during MAc_2_, individual transporters, individual sensors, and cellular constitution have changed in such a way as to make (*J*_E_–*J*_L_) more positive than during MAc_1_ and thereby reduce the magnitude of (ΔpH_i_)_2_ compared to (ΔpH_i_)_1_. **(C)** State of a consistent neuron during a second MAc challenge. We hypothesize that during MAc_2_, the net effect on (*J*_E_–*J*_L_) is the same as during MAc_1_ in panel A, although individual transporters, individual sensors, and cellular constitution may have changed. **(D)** State of a decompensated neuron during a second MAc challenge. We hypothesize that during MAc_2_, individual transporters, individual sensors, and cellular constitution have changed in such a way as to make (*J*_E_–*J*_L_) more negative than during MAc_1_ and thereby increase the magnitude of (ΔpH_i_)_2_ compared to (ΔpH_i_)_1_. The symbols have the same meanings, as detailed in the previous figures. The thickness of the arrows representing *J*_E_ and *J*_L_ and the thickness of the lines surrounding “Sensors” reflect the relative functional activities. The numbers of intracellular light-green “plus” symbols and red “minus” symbols reflect the degree of stimulation or inhibition by the Sensors and/or Constitution. Although we show equal numbers of intracellular light-green “plus” symbols and red “minus” symbols, it is really some combination of the two that reflects the relative degrees of transporter stimulation/inhibition by “Sensors” and/or “Constitution.”

Figure 12 presents cellular models of adaptation, consistency, and decompensation. In each case, the intracellular bright green “plus” boxes indicate stimulation of acid extrusion via some combination of the three factors: an increase in the number of transporters at the cell surface, an increase in the functional activity of extracellular sensors to increase *J*_E_, and changes in the cellular constitution that increase the unitary activity of acid extruders. The red “minus” boxes indicate the opposite for acid loading. Note that, in our cellular models, increases in *J*_E_ and decreases in *J*_L_ are interchangeable because they could produce similar changes in Δ(*J*_E_–*J*_L_). For simplicity, we show equal numbers of “plus” and “minus” boxes. In Figure 12, panels A_1_, A_2_, and A_3_ are identical.

#### Adaptation

Figures 12A1, B illustrate our model for how the three factors conspire to produce Δ(*J*_E_–*J*_L_) that is less negative during MAc_2_ than during the earlier MAc_1_. As implied by the presentation in the previous paragraph, a cell could achieve adaptation by increasing *J*_E_ from MAc_1_ to MAc_2_ without any change (or even a smaller decrease) in *J*_L_, or a decrease in *J*_L_ without any change (or even a smaller increase) in *J*_E_. In any case, MAc_2_ elicits a smaller pH_i_ decrease than MAc_1_—adaptation.

#### Consistency

Figures 12A2, C illustrate our model, showing how overall (*J*_E_–*J*_L_)—regardless of changes in individual components of *J*_E_ and *J*_L_—remains approximately the same during MAc_2_, as during MAc_1_ so that (ΔpH_i_)_2_ ≅ (ΔpH_i_)_1_,—consistency.

#### Decompensation

Figures 12A3, D illustrate our model, showing how overall (*J*_E_–*J*_L_)—regardless of changes in individual components of *J*_E_ and *J*_L_—decreases during MAc_2_ compared to MAc_1_. As a result, MAc_2_ elicits a larger pH_i_ decrease than MAc_1_,—decompensation.

### pAc_2_ and pMet↓_2_

**FIGURE 13:**
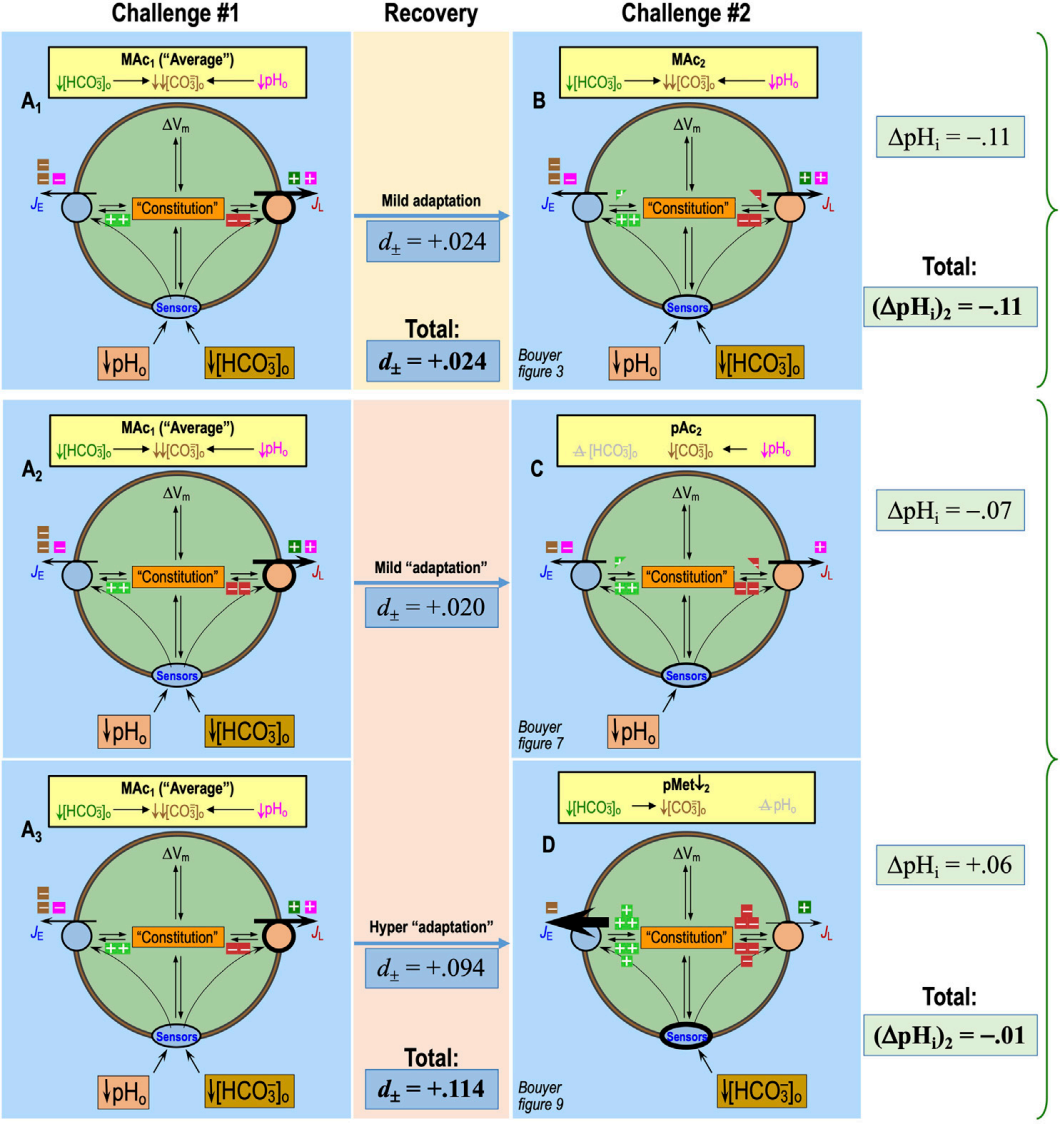
Model of the mechanism of non-additivity in MAc_1_-treated neurons: pAc_2_ + pMet↓_2_ >> MAc_2_. We hypothesize that MAc_1_ produces the usual initial percent inhibition (extracellular brown or magenta “minus” symbols) or stimulation (extracellular dark-green or magenta “plus” symbols) of each transporter (see Figure 5E) and sensor (see Figure 6) and generates an average-sized pH_i_ decrease (see Figure 8). After the recovery period, we expose the cell either to MAc_2_ panels **(A**_**1**_ **–B)**, pAc_2_ panels **(A**_**2**_ **–C)**, or pMet↓_2_ panels **(A**_**3**_ **–D)**. The different behavior responses during challenge #2 would reflect differences in (1) transporter numbers, (2) sensor numbers, and (3) cellular constitution (which would influence intrinsic transporter and sensor activity). The symbols have the same meanings as detailed in the previous figures. The thickness of the arrows representing *J*_E_ (rate of acid loading from all sources) and *J*_L_ (rate of acid extrusion from all sources) and the thickness of the lines surrounding “Sensors” reflect the relative functional activities (i.e., product of the protein number and intrinsic activity per protein). Although we show equal numbers of intracellular light-green “plus” symbols and red “minus” symbols, it is really a combination of the two that reflects the relative degrees of transporter stimulation/inhibition by “Sensors” and/or “Constitution.” The *d*_±_ values are from *table 2* of Bouyer et al. (2024). The “ΔpH_i_” values in light-green boxes refer to challenge #2 and are obtained from the *figure 3* panel **(B)**, *figure 7* panel **(C)**, and *figure 9* panel **(D)** of the same paper. The bolded “Total” values—either *d*_±_ or (ΔpH_i_)_2_—are either the same as for the single respective values in panel B or the sum of the two respective values for panels C and D. A_1_–A_3_, cell state during challenge #1. A_1_, A_2_, and A_3_ are identical. MAc_1_ produces a negative shift in Δ(*J*_E_–*J*_L_) and, therefore, a pH_i_ decrease, which is greater in magnitude than in panels B, C, or D. **(B)** Average response to MAc_2_. We hypothesize that some combination of increased *J*_E_ and decreased *J*_L_ produces a modestly negative Δ(*J*_E_–*J*_L_) that is smaller in magnitude than during MAc_1_. As a result, Δ(pH_i_)_2/MAc_ has a smaller magnitude than (ΔpH_i_)_1/MAc_—a mild adaptation. **(C)** Average response to pAc_2_. We hypothesize that some combination of increased *J*_E_ and decreased *J*_L_ produces a modestly negative Δ(*J*_E_–*J*_L_) that—as is the case in panel B—is smaller in magnitude than during MAc_1_. As a result, Δ(pH_i_)_2/pAc_ has a smaller magnitude than (ΔpH_i_)_1/MAc_—a mild “adaptation.” **(D)** Average response to pMet↓_2_. We hypothesize that some combination of increased *J*_E_ and decreased *J*_L_ produces a massively positive Δ(*J*_E_–*J*_L_). As a result, Δ(pH_i_)_2/pMet↓_ is frankly positive—a “hyper adaptation.”

In our presentation of Figure 11, we observed that, in a population of naïve neurons, the sum of ΔpH_i_ values in pAc_1_ and pMet↓_1_ is approximately equal to MAc_1_. Figure 13 illustrates an analogous analysis of pAc and pMet↓ but for neurons previously challenged in period #1 with MAc.

#### Non-additivity of pAc_2_ and pMet↓_2_ during MAc–MAc

Figures 13A1, B show cellular models of the average MAc–MAc data in *figure 3* of Bouyer et al. (2024), who reported a mild decrease in ΔpH_i_ from MAc_1_ (−0.14) to MAc_2_ (−0.11) in concert with a modestly positive *d*_±_ (+0.024). In Figure 13B, we model this mild adaptation by adding a “half-plus” for *J*_E_ and a “half-minus” for *J*_L_. Many combinations of *J*_E_ and *J*_L_ changes—produced by changes in the three factors^13^—could have elicited the required modest increase in (*J*_E_–*J*_L_).

Figures 13A2, C show models of the acidosis part of that MAc_2_. Because in *figure 7* of Bouyer et al. (2024), the (ΔpH_i_)_1/MAc_ value was smaller (−0.11) than that in their *figure 3* (−0.14), we interpret (ΔpH_i_)_2/pAc_ (−0.07) as representing the 0MAc-pAc equivalent of mild adaptation. The *d*_±_ value of *figure 7* (+0.020) was similar to that of *figure 3* (+0.024). Therefore, we model pAc_2_ in Figure 13C similarly to MAc_2_ in Figure 13B, with an addition of a half-plus to *J*_E_ and half-minus to *J*_L_. Our interpretation of Bouyer et al. (2024) data is that the stimulation of pH_o_ sensors by pAc_2_ provides all the impetus necessary to produce the usual changes observed during MAc_2_ of a MAc–MAc protocol; in other words, no input from HCO_3_^−^ sensors is necessary to account for (ΔpH_i_)_2/MAc_.

Figures 13A3, D show models of the ↓[HCO_3_^−^]_o_ part of that MAc_2_. A note of caution: *figure 9* of Bouyer et al. (2024) reports that (ΔpH_i_)_1/MAc_ was smaller (−0.07) than both the population average (−0.11 in their *figure 1*) and the value reported in *figure 3* (−0.14). Nevertheless, (ΔpH_i_)_2_ during pMet↓_2_ is notably striking: an average pH_i_ increase of +0.06 with ∼87% of all neurons exhibiting a net pH_i_ increase during pMet↓_2_ of the MAc-pMet↓ protocol. Thus, we are dealing with a strong phenotype. Furthermore, we recall that in naïve neurons, pMet↓_1_ produced the smallest recorded average pH_i_ decrease (−0.04) and nearly 40% of the neurons underwent a frank pH_i_ increase (see their *figure 8*). Returning to Bouyer’s *figure 9*, *d*_±_ is also strikingly large (0.094). Thus, in evaluating the data underlying [Fig F8] [Fig F9] through CD, we reach similar conclusions whether we sum population ΔpH_i_ values (−0.07 + +0.06 = −0.01) or population *d*_±_ values (+0.020 + +0.094 = +0.114): in other words, the sum of the parts in panels C and D is far greater than the overall result in panel B ((ΔpH_i_)_2_ = −0.11, *d*_±_ = +0.024). How is this discrepancy possible?

We propose that HC neurons, during MAc_2_ of a physiological MAc–MAc challenge, normally engage in coincidence detection involving two sets of acid–base sensors—one for extracellular H^+^ and another for extracellular HCO_3_^−^. When the two challenges arrive with approximate simultaneity, their respective signal transduction cascades have the effect of muting one another, especially muting the strong actions of decreased [HCO_3_^−^]_o_ during MAc_2_.

#### Extreme paradoxical behavior pMet↓_2_

In Figure 10, we proposed—knowing the pH_i_ responses to pMet↓_1_ and pMet↓_2_—that a small subset (∼10%) of naïve rat HC neurons respond to MAc_1_ with an unbalanced activation of RPTPγ or RPTPζ, resulting in a paradoxical pH_i_ increase.

We propose that, also in naïve neurons, pMet↓_1_—without accompanying muting contributed by decreased pH_o_ in MAc_1_—produces an even more unbalanced net stimulation of acid extrusion. In nearly 40% of the population, this results in a paradoxical pH_i_ increase.

Finally, in HC neurons primed with an MAc_1_ challenge and then allowed to recover, the subsequent exposure to pMet↓_2_ produces the greatest unbalanced net increase in (*J*_E_–*J*_L_) such that ∼87% of Bouyer’s HC neurons exhibited a paradoxical pH_i_ increase. During/after MAc_1_, the neuron does not “know” what the experimenter intends for the second challenge. MAc_1_ and the subsequent recovery period must set the stage for the truly remarkable alkalinizing response (i.e., ↑pH_i_) to pMet↓_2_ in Bouyer’s *figure 9*. Bouyer likened this phenomenon to placing 100 glasses of room-temperature water into a functioning refrigerator, only to find that, after their removal, 87 glasses had warmed up (!).

#### MAc_2_ in a normal MAc–MAc protocol

**FIGURE 14:**
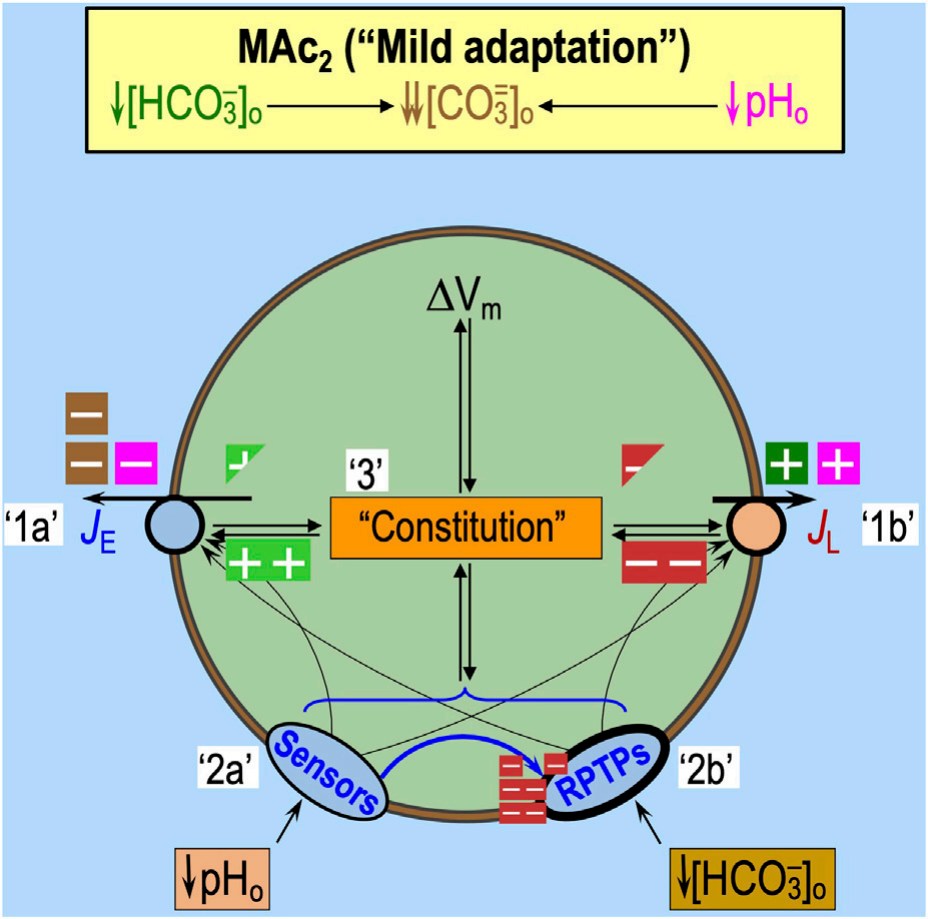
Revised mechanistic model of mild adaptation during MAc_2_: coincidence detection. Inspired by the unique predictions in Figure 13D, we present this general model. We envisage that the response to MAc_2_ represents more than the simple additivity of low-pH_o_ and low-[HCO_3_^−^]_o_ stimuli, as depicted in Figure 13B. In this figure, we split the generic ↓pH_o_/↓[HCO_3_^−^]_o_ “Sensors” icon into separate sensors for ↓pH_o_ (‘2a’) and ↓[HCO_3_^−^]_o_ (‘2b’). Although we retain the ability of the now-separate sensors—acting in parallel—to stimulate acid extrusion (‘1a’) and inhibit acid loading (‘1b’) and interact with “Constitution” (‘3’), we now introduce a new concept: the ↓pH_o_ sensors (‘2a’) must normally act during MAc_2_ to antagonize the ↓[HCO_3_^−^]_o_ sensor (‘2b’). Thus, we envisage that the sensors act in three ways: (1) pre-conditioned by MAc_1_, the ↓[HCO_3_^−^]_o_ sensors (e.g., RPTPγ and/or RPTPζ) are poised to produce—via actions of *J*_E_, *J*_L_, and Constitution—a massive increase in (*J*_E_–*J*_L_), which, by itself, would produce a paradoxical pH_i_ increase, as modeled in Figure 13D. Perhaps also pre-conditioned by MAc_1_, the ↓pH_o_ sensors (e.g., GPR68) have two effects. (2) Parallel increase—via actions of *J*_E_, *J*_L_, and Constitution—in (*J*_E_–*J*_L_). (3) Massive inhibition of the response of the RPTPs to the low-[HCO_3_^−^]_o_ stimulus. This model is a great oversimplification. The *J*_E_ (rate of acid loading from all sources) and *J*_L_ (rate of acid extrusion from all sources) icons represent a multitude of individual transporters. The sensors, although split into separate detectors of ↓pH_o_ and ↓[HCO_3_^−^]_o_, could represent multiple examples of each (see Figure 6). Constitution we defined as “the collection of all ion-concentration, metabolic, and signaling properties.” We envisage all of the individual effects to be time-dependent, both in terms of activation and deactivation (e.g., persistence). Dependencies of transporters, sensors, and enzymes on the concentrations of their relevant substrates/ligands are almost certainly nonlinear. When two arrows point at a target, the effects could be sub-additive, simply additive, or supra-additive (i.e., synergistic). The proposed inhibitory effect of the ↓pH_o_ on the ↓[HCO_3_^−^]_o_ sensors is an example of antagonism. Assembling all of these complexities into a useful model is a task for mathematical modeling. Although we show equal numbers of intracellular light-green “plus” symbols and red “minus” symbols, it is really some combination of the two that reflects the relative degrees of transporter stimulation/inhibition by “Sensors” and/or “Constitution.”

During MAc–MAc, one can view MAc_2_—in a neuron that has already been preconditioned by MAc_1_—as being pMet↓_2_ (see Figure 13D) supplemented with pAc_2_. We propose that the pAc_2_ component—acting via ↓pH_o_ sensors like GPR68, ASICs, and TASKs (see Figure 6)—nullifies most of the alkalinizing effects of pMet↓_2_ (Figure 14), an example of antagonism. Such H^+^-induced nullification may also occur to some extent in naïve neurons, and its variability could underlie some of the “state” variability observed in naïve neurons, as well as in those first challenged with MAc_1_ and later subjected to MAc_2_. See the legend of Figure 14 for a consideration of how our cartoon model is an oversimplification of the complexities of physiology. Refer to the section on mathematical modeling^14^ for suggestions on how to address these complexities.

### Parameters’ potentially governing behavior

Not addressed in the studies of Salameh et al. (2014) and Bouyer et al. (2024) are several important unresolved questions regarding the duration of events required for establishing behaviors:
• Challenge #1: How does the development of a behavior depend on challenge #1, particularly its:
  o nature (e.g., MAc_1_ vs. pMet↓_1_)?
  o intensity (e.g., degree of lowering pH_o_ or [HCO_3_^−^]_o_)?
  o duration?
• Recovery-period duration: How does the development of a particular behavior depend on the duration of the interlude period between challenges #1 and #2? The answer could depend on
  o Preceding challenge #1 (nature, intensity, and duration)
  o Challenge #2 (nature and intensity)
• Extinguishment: Over what duration of recovery period would the behavior-inducing effects of challenge #1 extinguish? The answer could depend on
  o Preceding challenge #1 (duration, nature, and intensity)
  o Challenge #2 (nature and intensity)

We suggest that a fruitful initial approach for addressing the abovementioned questions could be to use the MAc-pMet↓ protocol as a test case because it produces the most reproducible and remarkable responses. Recall that *figure 9b* in the study of Bouyer et al. (2024) shows that ∼87% of neurons subjected to this protocol exhibit a paradoxical pH_i_ increase.

It is of interest that, in the study of Bouyer et al. (2024) and the PhD dissertation of Taki (2024), the initial MAc_1_-induced pH_i_ decrease (indicative of a negatively shifted [*J*_E_–*J*_L_]) was not followed by a delayed pH_i_ increase (reminiscent of adaptation) or pH_i_ decrease (reminiscent of decompensation) during MAc_1_. Thus, we can conclude that either (1) the duration of challenge #1 (e.g., 7 min in the Taki study) was too brief for the development of a secondary change in (*J*_E_–*J*_L_) or (2) the removal of challenge #1 is necessary for the development of the behavior observed during challenge #2.

Although we have primarily focused on acid–base parameters as potential modulators of behavior, other environmental factors—metabotropic signaling molecules and the ionic milieu (e.g., [K^+^]_o_)—also could also be in play.

#### Summary

At the population level, the behavior evidenced during MAc_2_ is quite different from the simple sum of pAc_2_ and pMet↓_2_. Behavior could depend on the nature, intensity, and duration of challenge #1, as well as the duration of the recovery period. The response to pMet↓_2_, perhaps mediated by RPTPγ/*ζ*, is extremely powerful, capable of producing rather consistent paradoxical increases in pH_i_.

## Impact on extracellular buffering

### Resistant state and adaptation

Both a relatively resistant state and an adaptive behavior could be the appropriate “selfish” response of neurons, for which a relatively large acidic pH_i_ shift would have a negative impact on the physiological role of analogous neurons in an intact brain. Such hypothetical neurons—those especially critical under a particular set of circumstances—may be programmed to reduce the magnitude of acidic shifts using the strategies outlined above. The price to pay for such selfishness is that the cell’s small negative (*J*_E_–*J*_L_) necessarily results in the extrusion of acid into the extracellular space (see Figure 9C and Figure 12B), which lays an extra low-pH_o_ burden on neighboring cells.

### Average state and consistency

Both an average^10^ state and a consistent behavior could be the appropriate “unselfish” response of neurons, for which an acidic pH_i_ shift would have a limited impact on the physiological role of an analogous neuron in an intact brain. By allowing themselves to acidify moderately during MAc_1_ and/or to acidify no more during MAc_2_ than during MAc_1_, such neurons perform an important function by buffering extracellular acid and reducing extracellular acid loads experienced by neighboring cells.

### Sensitive state and decompensation

Both a relatively sensitive state and a decompensating behavior could be the appropriate “altruistic” response of neurons, for which a relatively severe acidic pH_i_ shift would have limited impact on the physiological role of an analogous neuron in an intact brain. By allowing themselves to acidify to a relatively large degree during MAc_1_, and/or acidifying more during MAc_2_ than during MAc_1_, these altruistic neurons buffer disproportionately greater fractions of extracellular acid loads and thereby spare their neighboring cells.

### Astrocytes vs. neurons in the CNS

Although we have focused on neurons, it is interesting to recall that Salameh et al. (2014) found that, during a MAc–MAc protocol, ΔpH_i_/ΔpH_o_ is consistently greater (by nearly 50%) for astrocytes than for neurons, both in cultures from the hippocampus and medullary raphé and both for MAc_1_ and MAc_2_. On the other hand, intrinsic intracellular buffering power (β_I_; see Thornell et al. (2025) has the opposite pattern for the two cell types. For cultured astrocytes from rat HC, β_I_ is only ∼10 mM/pH at pH_i_ 7.0 in one study (Bevensee et al., 1997), whereas β_I_ of neurons acutely isolated from rat HC is much higher, ∼15 mM/pH at pH_i_ 7.0 in another study (Bevensee et al., 1996). Thus, if we consider only β_I_, although their ΔpH_i_ may be 50% greater, HC astrocytes would take up about the same amount of acid per unit volume of cytosol as neurons. On the other hand, total intracellular buffering power (β_T_) is the sum of β_I_ and the open-system CO_2_/HCO_3_^−^ buffering power (β_open_), with the latter increasing exponentially^15^ with pH_i_ and being the same in all cells. Thus, at relatively low pH_i_ values, β_T_ would be modestly lower in astrocytes than in neurons, and astrocytes (with a 50% greater ΔpH_i_) would buffer modestly more acid than neurons. At relatively high pH_i_ values, β_open_ would overwhelm β_I_, and thus, β_T_ would be rather similar in the two cell types; in this case, astrocytes would buffer much more acid than neurons. Because astrocytes can undergo rather large pH_i_ changes and buffer more acid than neurons on average, we could view them as being altruistic compared to neurons.

## Variability among neuronal responses

### pH sensitivity of neurons

Changes in pH_o_ or pH_i_ can affect a wide range of electrophysiological properties because of the pH_i_/pH_o_ sensitivity of virtually every neuronal component—including channels, receptors, transporters, enzymes (including those involved in neurotransmitter metabolism), and cytoskeletal elements. Thus, one would expect that inappropriate pH_i_ changes could lead to CNS pathology. It is generally believed that high neuronal pH_i_ is pro-epileptogenic (Hentschke et al., 2006; Jacobs et al., 2008; Sinning et al., 2011). Chesler and Kraig (1987), Chesler and Kraig (1989) and Ransom (2000) proposed a negative-feedback model, which was discussed and extended by Salameh et al. (2017). In this model, neuronal activity leads to an increase in [K^+^]_o_, causing depolarization-induced alkalinization (DIA) in astrocytes (Siebens and Boron, 1989a; Siebens and Boron, 1989b), that in turn would cause a fall in pH_o_—a local MAc—and, thus, a generalized reduction in neuronal excitability. We would expect that local MAc would lower neuronal pH_i_. Induced epileptiform activity lowers neuronal pH_i_, which recovers after the epileptiform activity ceases (Xiong et al., 2000; Raimondo et al., 2012). Although high pH_o_ is also considered to be pro-epileptogenic, chronic low-grade metabolic acidosis may contribute to the development of chronic epilepsy (Yuen, 2006).

Based on the above discussion, one might have expected the distribution of neuronal pH_i_ values to be within a relatively narrow range. However, a striking characteristic of mammalian HC neurons, freshly dissociated or cultured, is an unusually wide range of resting pH_i_ values (Schwiening and Boron, 1994; Baxter and Church, 1996; Bevensee et al., 1996; Smith et al., 1998) compared to other cell types. Moreover, our laboratory has identified a wide range of ΔpH_i_ responses to MAc (Bouyer et al., 2004; Salameh et al., 2014; 2017) and twin MAc–MAc challenges (Salameh et al., 2014; 2017). Bouyer et al. (2024) study confirms this diversity of responses to MAc (state) and MAc–MAc (behavior) and extends both aspects of diversity to the artificial acid–base disturbances pAc and pMet↓, alone and in combination with MAc.

### Origin of pH_i_ diversity in neurons

We have already presented hypotheses to address the molecular mechanisms underlying the diversity of state and behavior. We now ask, at a higher level, what is responsible for the aforementioned diversity? We offer four possibilities that are not mutually exclusive.

(1) Some of the diversity is unphysiological. For example, the above-cited studies show that the broad range of initial pH_i_ values is greater in the absence than in the presence of CO_2_/HCO_3_^−^ (which would presumably enable the full complement of pH_i_-regulatory mechanisms). It is possible that the range would be narrower still if we were to study the neurons *in vivo*, where they would be under the potential influence of metabotropic signaling and other influences from neighboring cells in a three-dimensional arrangement.
(2) Some of the acid–base diversity represents a diversity of neuronal subtypes, with each subtype, as studied in primary culture, having its own range of expressions for each relevant protein.
(3) Some of the diversity is intrinsic to neuronal physiology (nature), at least in primary culture, reflecting apparently stochastic differences in the numbers and localization of various proteins.
(4) Some of the diversity depends on the history of individual neurons (nurture), including differences in the acid–base microenvironment, patterns of neuronal activity, and other environmental parameters (e.g., cell–cell contacts) for these cells in primary culture.

Thus, each neuron in culture could have a set of properties that depends on neuronal subtype, stochastic variations in protein numbers/localization within that subtype (nature), and differences in neuronal history (nurture). Together, these factors could establish a constitution that determines how a particular neuron responds to one acid–base challenge (state) or a sequence of them (behavior).

### Impact on cell function

A teleological question that arises is why should such diversity exist? One advantage of diversity could be to increase the probability that enough neurons in a circuit can withstand periodic acid–base challenges of various types. Another could be that neurons with different electrophysiological properties (and the underlying cohort of ion channels and other proteins, each with its own pH_i_ sensitivity) could be more electrically stable with a resistant vs. sensitive state or with an adaptive vs. consistent vs. decompensatory behavior.

## Discussion

### Major conclusions

Regarding the experiments with OOE solutions, we believe that the main conclusions of the paper by Bouyer et al. (2024) can be summarized as follows.

#### “State” during challenge #1

In a population of naïve rat HC neurons, the effects of pAc_1_ and pMet↓_1_ on pH_i_—assessed as (ΔpH_i_)_1_—are approximately additive. In other words, in naïve HC neurons, whole MAc_1_ is approximately the sum of its parts (see Figure 11).

#### Acid–base sensors

The abovementioned results lead to the conclusion that rat HC neurons have separate sensors that detect (1) a decrease in pH_o_ and (2) a decrease in [HCO_3_^−^]_o_ (see Figure 6).

#### “Behavior” when challenge #1 is MAc_1_

In a population of neurons that has already experienced MAc_1_ followed by a recovery period, the subsequent effects of pAc_2_ and pMet↓_2_—as assessed by either (ΔpH_i_)_2_ or *d*_±_—are decidedly not additive (see Figure 13).

#### Coincidence detection

The abovementioned result leads us to conclude that—for this protocol, which spans MAc_1_ and a recovery period—pAc_2_ and pMet↓_2_ challenges must arrive at approximately the same time to reproduce the physiological effects of MAc_2_ (see Figure 14).

### Molecular mechanism

Mouse HC neurons express both RPTPγ and RPTPζ (Lorenzetto et al., 2014; Taki et al., 2024). Based on the PhD dissertation of Taki (2024), who examined the effect of knocking out RPTPζ in MAc–MAc and RAc–RAc protocols on mouse HC neurons, and the work of Zhou et al. (2016), who examined the effect of knocking out RPTPγ in renal proximal tubules, we propose that the most likely HCO_3_^−^ sensor(s) in the experiments of Bouyer et al. (2024) are some combination of RPTPγ and RPTPζ.

In addition, we urge additional experiments that further probe the molecular mechanisms underlying state and behavior and suggest an extension of the studies to include (1) the duration and intensity of the first challenge, (2) the duration of the recovery period, and (3) additional acid–base challenges that involve both equilibrated solutions (i.e., RAc, MAlk, and RAlk) and OOE solutions (pAlk, pMet↑, pResp↓, and pResp↑).

### Mathematical modeling

Aside from a call for more wet-laboratory data, we urge the development of mathematical models—the counterparts of the qualitative models described with words and cartoons in the present paper—that could assist in the interpretation of experiments like those in the research paper by Bouyer et al. (2024). Boron and De Weer (1976) developed the first mathematical model of pH_i_ regulation, a compartmental model that embodies the principles of the fundamental law of pH_i_ regulation in Equation 10. Occhipinti et al. (2020), as part of the *Physiome* journal (which is part of the broader Physiome Project), wrote a retrospective of the BDW model that includes clarifications and updates, access to online implementations, and a summary of several post-BDW models of pH_i_ regulation.

The set of two ordinary differential equations in the BDW model includes only one component of *J*_L_ (an H^+^ pump, the rate of which varies with [H^+^]_i_—and thus time—according to a fixed rate constant) and a single component of *J*_E_ (an HCO_3_^−^ leak, the rate of which varies with [HCO_3_^−^]_i_—and thus pH_i_ and time—according to a fixed HCO_3_^−^ permeability). Although it would be straightforward to incorporate additional components of *J*_E_ (i.e., *J*_E1_, *J*_E2_, … ) and *J*_L_ (i.e., *J*_L1_, *J*_L2_, … ), imbuing these components (e.g., variants of NBCn1) with realistic estimates of sensitivity to pH_i_ and pH_o_, as well as their respective substrates, would require major—but valuable—investments from funding agencies. The same applies to acid–base sensors and the broader issue of “cellular constitution,” which would describe diverse influences ranging from ion concentrations to signal transduction pathways.

Figure 14 is a cartoon model of how interactions among (1) transporters, (2) sensors, and (3) cellular constitution could account for the results of the recent study by Bouyer et al. (2024). As noted in the figure legend, the cartoon is greatly oversimplified: each transporter icon represents a multitude of individual proteins. The ↓pH_o_ and ↓[HCO_3_^−^]_o_ sensors could represent multiple examples of each (see Figure 6). We defined constitution as “the collection of all ion-concentration, metabolic, and signaling properties.” All of the interconnected components vary with time during—and after—and acid–base challenge. Their dependencies on concentrations of their relevant substrates/ligands are almost certainly nonlinear. Effects could have varying degrees of additivity or antagonism.

A goal of programs such as The Physiome is to develop modular mathematical components for each transporter and signaling pathway, assemble the components into various model cells, and inform the models from experiments like those reported in the research paper by Bouyer et al. (2024). We envision the development of such sophisticated models—the quantitative versions of those qualitative models in the present paper—and using them to interpret the research paper by Bouyer et al. (2024) and design future experiments.

### Limitations to the model(s)

We begin by acknowledging the principle that “all models are wrong, but some are useful”—the first part of which is articulated by the British statistician Box (1976), who also emphasized the concept of “useful.”

The word and cartoon models presented in this paper are based on the fundamental law of pH_i_ regulation (see Equation 10), which is mathematically expressed as follows:

$$\frac{d{\text{pH}}_{i}}{\text{dt}}=\frac{\rho }{\beta }\cdot \left(J_{E}-J_{L}\right)\;.$$ (11)

This equation is analogous to the principle of continuity in physiology^16^ or fluid mechanics, which, in turn, is based on the conservation of mass. Thus, the basic model must be correct, at least at the integrative level of classical physics and chemistry.

The theoretical cartoon models presented in this paper could be tested by employing more sophisticated mathematical modeling approaches than that explained in Equation 10. These approaches could resort to “compartmental” models, treating the cell and the extracellular fluid as uniform compartments with instantaneous mixing (i.e., ignoring a detailed spatial description of the cell geometry and its effects on solute diffusion). More complex approaches could resort to 3D (“distributed”) reaction-diffusion models, in which one accounts for the diffusion (or transport) of solutes in 3D space/time, as well as the chemical reactions that occur in parallel 3D space/time. For example, such models can attempt to account for unconvected layers that surround a cell and how geometry impacts the time courses of solute concentrations. In either case, the modeler formulates the problem using differential equations and solves these using various numerical methods. Assumptions—and opportunities for error—abound at each conceptual step.

Limitations also arise in the complexity of the biological system and the oversimplifications by which we estimate individual terms, even in the relatively simplest of approaches (e.g., a compartmental system):

#### Surface-to-volume ratio (ρ)

Although distributed mathematical models can describe complex cell geometries explicitly, they face increasing computational challenges when solving numerically the resulting (partial) differential equations. Generally, whenever possible, modelers overcome this challenge by simplifying cellular geometry, assuming that a cell has a simple geometric shape (e.g., a sphere or a cylinder). In the case of the oocyte models of Somersalo et al. (2012) and Occhipinti et al. (2014), the authors took advantage of the oocyte’s being a spherical cell and further simplified the model by assuming spherical radial symmetry (i.e., only the distance from the cell center influences solute transients). Occhipinti et al. (2014) incorporated an amplification of the surface area to accommodate microvilli. Even so, the volumes and surface areas of living cells are not precisely known, and they can change with time.

#### Buffering power (β)

Modelers might break buffering into two components: open-system buffering power (due to a solute like CO_2_ or NH_3_ that can equilibrate across the cell membrane) and intrinsic buffering power (Boron, 1977). As discussed by Thornell et al. (2025), the intrinsic buffering power (βI) of the cytosol comprises chemical buffering (due to classic acid–base equilibria), biochemical buffering (due to other reactions that consume/generate H^+^), and organellar buffering (due to the movement of H^+^ equivalents across organellar membranes). Although it may be reasonably straightforward to account for open-system buffering, βI is, in principle, extraordinarily complicated because myriad components contribute to it, and this could change with time and metabolic state. In addition, intrinsic buffering power is pH_i_-sensitive—although it is possible to measure this, as first done by Boron (1977). The modeler might assume a constant/fixed βI, a constant pH_i_-dependent βI, or—as done by Somersalo et al. (2012) and Occhipinti et al. (2014)—represent βI with a single chemical buffer pair (HA ⇌ H^+^ + A^–^) using a pK and total concentration chosen to mimic the cell buffering power. All of the above represent limitations to models.

#### Acid extrusion (*J*_E_) and acid loading (*J*_L_)

As noted in our discussion during the introduction of Equation 10, the overall *J*_E_ and *J*_L_ each comprise a multitude of different transporters (see Figure 3), each with a distinct set of kinetic parameters. To the best of our knowledge, not even one acid–base transporter is fully described kinetically. Thus, modelers are left to estimate the parameter values—a further limitation to quantitative models. The numbers and activities of the individual *J*_E_/*J*_L_ components are likely to change with time and acid–base challenges like those discussed in the present paper—further limiting models.

#### Extracellular acid–base sensors

In Figure 6, we introduced several classes of extracellular acid–base sensors, of which we know of several pH_o_-sensitive GPCRs (for review, see Thornell et al., 2025), pH-sensitive ion channels like ASICs and TASKs, and two CO_2_/HCO_3_^−^-sensitive RPTPs, each with several variants. GPCRs and RPTPs could each modulate individual acid–base transporters, probably as the result of complex signal transduction cascades. GPCRs and RPTPs could also modulate pH_o_-sensitive channels like ASICs and TASKs and a myriad of other cellular processes that constitute cellular constitution. We do not fully understand the role of any one of the above in modulating pH_i_ homeostasis. All of the above uncertainty contributes to the limitations to models.

#### Cellular constitution

We defined cellular constitution as “the collection of all [intracellular] ion-concentration, metabolic, and signaling properties” that can directly impact (1) the transporters directly responsible for *J*_E_ and *J*_L_ and (2) extracellular acid–base sensors. This catch-all grouping of constantly changing (1) small inorganic and organic molecules and (2) peptides and other polymers (including proteins and nucleic acids) will be a major challenge to characterize. Liquid–liquid phase separations may be the loci of many important biochemical processes. The extensive uncertainty about all of the above processes contributes to model limitations.

Although the preceding discussion may seem discouraging, we are optimistic that—over the decades—a continuous effort by cellular physiologists will enable them to develop and inform models that—although “wrong”—become increasingly more “useful” in interpreting data and formulating further hypotheses (to be tested experimentally).

## Data Availability

Publicly available datasets were analyzed in this study. These data can be found here: in the companion paper.

## Footnotes

1 By acute, we loosely mean a few seconds to minutes. Over a period of hours, genomic responses to acid–base disturbances could involve other signaling pathways.

2 See the section titled “State & Behavior.”

3 We refer to figures, tables, equations, and named solutions in the 2024 Bouyer paper in italics, using all lowercase letters.

4 Bouyer et al. (2024) chose this pMet↓ nomenclature to make room for a future “pMet↑” solution, in which [HCO_3_^−^]_o_ would increase at a fixed [CO_2_]_o_ and pH_o_.

5 As embodied in Equation, ρ and β influence the rate at which pH_i_ changes during extracellular acid–base disturbances but not the direction of the pH_i_ change or even steady-state pH_i_.

6 In the Discussion of that paper, see the section titled “Effects of acid–base challenges on [CO_3_^=^]_o_.”

7 See “Determinants of neuronal state and behavior.”

8 Here, we consider acute effects with a duration of minutes. If the challenges were to persist long enough, changes in protein synthesis could affect the quantity and identity of expressed proteins.

9 See “Molecular basis … ”).

10 We will use “average cell” in two closely related ways to denote a cell with a ΔpH_i_, that is (1) the mean value for the population and (2) near the boundary between “resistant” and “sensitive” states, as shown in Figure 8.

11 See inline heading “Paradoxical responses.”

12 Note that we make no statement about how the challenge affects the magnitudes or even the directions of the shifts in *J*_E_ vs. pH_i_ or *J*_L_ vs. pH_i_ (see Figures 8B, C) or the time courses of these shifts. We only require that the negative Δ(*J*_E_–*J*_L_), integrated over the period of the MAc challenge, produces (in this case) a “moderate” net intracellular acid load, resulting in a moderate decrease in pH_i_. For example, one could imagine a situation in which MAc caused *J*_L_ to paradoxically increase but caused *J*_E_ to increase even more. Because Δ(*J*_E_–*J*_L_) < 0, pH_i_ would still decrease.

13 (1) Identity and numbers of acid–base transporters in the plasma membrane, (2) extracellular acid–base sensors, and (3) cellular constitution. See “Molecular basis of effects of extracellular acid–base disturbances.”

14 See “Discussion” > “Mathematical modeling.”

15 β_open_ = 2.3 × [HCO_3_^−^]_i_ = 2.3 × [HCO_3_^−^]_o_ × 10^(pH_i_–pHo)^.

16 For example, the rate at which the volume of the chamber changes is determined by the difference between the blood inflow and blood outflow. If chamber volume is constant, then inflow must equal outflow.

## References

[B1] Alvarez de la R. Krueger S. R. Kolar A. Shao D. Fitzsimonds R. M. Canessa C. M. (2003). Distribution, subcellular localization and ontogeny of ASIC1 in the mammalian central nervous system. J. Physiology 546, 77–87. 10.1113/jphysiol.2002.030692 PMC2342460 12509480

[B2] Baxter K. A. Church J. (1996). Characterization of acid extrusion mechanisms in cultured fetal rat hippocampal neurones. J. Physiol. (Lond.) 493 (Pt 2), 457–470. 10.1113/jphysiol.1996.sp021396 8782109 PMC1158930

[B3] Bevensee M. O. Bashi E. Schlue W. R. Boyarsky G. Boron W. F. (1999). Shrinkage-induced activation of Na^+^/H^+^ exchange in rat renal mesangial cells. Am. J. Physiol. 276, C674–C683. 10.1152/ajpcell.1999.276.3.C674 10069995

[B4] Bevensee M. O. Boron W. F. (2013). “Control of intracellular pH,” in Seldin and giebisch’s the kidney: physiology and pathophysiology (Academic Press), 1773–1835.

[B5] Bevensee M. O. Cummins T. R. Haddad G. G. Boron W. F. Boyarsky G. (1996). pH regulation in single CA1 neurons acutely isolated from the hippocampi of immature and mature rats. J. Physiol. (Lond) 494, 315–328. 10.1113/jphysiol.1996.sp021494 8841993 PMC1160636

[B6] Bevensee M. O. Weed R. A. Boron W. F. (1997). Intracellular pH regulation in cultured astrocytes from rat hippocampus. I. Role of HCO₃^−^ . J. Gen. Physiol. 110, 453–465. 10.1085/jgp.110.4.453 9379175 PMC2229379

[B7] Boron W. F. (1977). Intracellular pH transients in giant barnacle muscle fibers. Am. J. Physiol. 233, C61–C73. 10.1152/ajpcell.1977.233.3.C61 20782

[B8] Boron W. F. (1985). Intracellular pH-regulating mechanism of the squid axon. Relation between the external Na^+^ and HCO_3_ ^−^ dependences. J. Gen. Physiol. 85, 325–345. 10.1085/jgp.85.3.325 2985734 PMC2215796

[B9] Boron W. F. (2004). Regulation of intracellular pH. Adv. Physiol. Educ. 28, 160–179. 10.1152/advan.00045.2004 15545345

[B10] Boron W. F. (2017). “Acid-base physiology,” in Medical physiology: a cellular and molecular approach. Editors Boron W. F. Boulpaep E. L. (Philadelphia, PA: Saunders Elsevier), 628–646.

[B11] Boron W. F. Boulpaep E. L. (1983). Intracellular pH regulation in the renal proximal tubule of the salamander. Basolateral HCO₃^−^ transport. J. Gen. Physiol. 81, 53–94. 10.1085/jgp.81.1.53 6833997 PMC2215562

[B12] Boron W. F. De Weer P. (1976). Intracellular pH transients in squid giant axons caused by CO2, NH3, and metabolic inhibitors. J. Gen. Physiol. 67, 91–112. 10.1085/jgp.67.1.91 1460 PMC2214912

[B13] Boron W. F. Knakal R. C. (1989). Intracellular pH-regulating mechanism of the squid axon. Interaction between DNDS and extracellular Na^+^ and HCO₃^−^ . J. Gen. Physiol. 93, 123–150. 10.1085/jgp.93.1.123 2915212 PMC2216203

[B14] Boron W. F. Knakal R. C. (1992). Na^+^-dependent Cl^−^-HCO₃^−^ exchange in the squid axon. Dependence on extracellular pH. J. Gen. Physiol. 99, 817–837. 10.1085/jgp.99.5.817 1607854 PMC2216616

[B15] Boron W. F. Russell J. M. (1983). Stoichiometry and ion dependencies of the intracellular-pH-regulating mechanism in squid giant axons. J. Gen. Physiol. 81, 373–399. 10.1085/jgp.81.3.373 6842177 PMC2215574

[B16] Bouyer P. Bradley S. R. Zhao J. Wang W. Richerson G. B. Boron W. F. (2004). Effect of extracellular acid-base disturbances on the intracellular pH of neurones cultured from rat medullary raphe or hippocampus. J. Physiol. (Lond.) 559, 85–101. 10.1113/jphysiol.2004.067793 15194736 PMC1665070

[B17] Bouyer P. Zhou Y. Boron W. F. (2003). An increase in intracellular calcium concentration that is induced by basolateral CO_2_ in rabbit renal proximal tubule. Am. J. Physiol. Ren. Physiol. 285, F674–F687. 10.1152/ajprenal.00107.2003 12812914

[B18] Bouyer P. G. Salameh A. I. Zhou Y. Kolba T. N. Boron W. F. (2024). Effects of extracellular metabolic acidosis and out-of-equilibrium CO₂/HCO₃^−^ solutions on intracellular pH in cultured rat hippocampal neurons. Front. Physiol. 15, 1434359. 10.3389/fphys.2024.1434359 39444753 PMC11496273

[B19] Box G. E. P. (1976). Science and statistics. J. Am. Stat. Assoc. 71, 791–799. 10.1080/01621459.1976.10480949

[B20] Brett C. L. Kelly T. Sheldon C. Church J. (2002). Regulation of Cl^−^/HCO_3_ ^−^ exchangers by cAMP-dependent protein kinase in adult rat hippocampal CA1 neurons. J. Physiol. (Lond.) 545, 837–853. 10.1113/jphysiol.2002.027235 12482890 PMC2290728

[B21] Chen J. Martinez J. Milner T. A. Buck J. Levin L. R. (2013). Neuronal expression of soluble adenylyl cyclase in the mammalian brain. Brain Res. 1518, 1–8. 10.1016/j.brainres.2013.04.027 23611875 PMC3679342

[B22] Chen Y. Cann M. J. Litvin T. N. Iourgenko V. Sinclair M. L. Levin L. R. (2000). Soluble adenylyl cyclase as an evolutionarily conserved bicarbonate sensor. Science 289, 625–628. 10.1126/science.289.5479.625 10915626

[B23] Chesler M. Kraig R. P. (1987). Intracellular pH of astrocytes increases rapidly with cortical stimulation. Am. J. Physiol. Regul. Integr. Comp. Physiol. 253, R666–R670. 10.1152/ajpregu.1987.253.4.R666 PMC2805720 3116863

[B24] Chesler M. Kraig R. P. (1989). Intracellular pH transients of mammalian astrocytes. J. Neurosci. 9, 2011–2019. 10.1523/JNEUROSCI.09-06-02011.1989 2723764 PMC2690820

[B25] Davis B. A. Hogan E. M. Boron W. F. (1994). Shrinkage-induced activation of Na^+^-H^+^ exchange in barnacle muscle fibers. Am. J. Physiology - Cell Physiology 266, C1744–C1753. 10.1152/ajpcell.1994.266.6.C1744 8023904

[B26] Hayashi N. Oohira A. Miyata S. (2005). Synaptic localization of receptor-type protein tyrosine phosphatase zeta/beta in the cerebral and hippocampal neurons of adult rats. Brain Res. 1050, 163–169. 10.1016/j.brainres.2005.05.047 15982644

[B27] Hentschke M. Wiemann M. Hentschke S. Kurth I. Hermans-Borgmeyer I. Seidenbecher T. (2006). Mice with a targeted disruption of the Cl^−^/HCO_3_ ^−^ exchanger AE3 display a reduced seizure threshold. Mol. Cell. Biol. 26, 182–191. 10.1128/MCB.26.1.182-191.2006 16354689 PMC1317631

[B28] Hogan E. M. Davis B. A. Boron W. F. (1997). Intracellular Cl^−^ dependence of Na-H exchange in barnacle muscle fibers under normotonic and hypertonic conditions. J. Gen. Physiol. 110, 629–639. 10.1085/jgp.110.5.629 9348333 PMC2229391

[B29] Horiguchi K. Higuchi M. Yoshida S. Nakakura T. Tateno K. Hasegawa R. (2014). Proton receptor GPR68 expression in dendritic-cell-like S100β-positive cells of rat anterior pituitary gland: GPR68 induces interleukin-6 gene expression in extracellular acidification. Cell Tissue Res. 358, 515–525. 10.1007/s00441-014-1958-x 25129106

[B30] Huang W. C. Swietach P. Vaughan-Jones R. D. Ansorge O. Glitsch M. D. (2008). Extracellular acidification elicits spatially and temporally distinct Ca^2+^ signals. Curr. Biol. 18, 781–785. 10.1016/j.cub.2008.04.049 18485712

[B31] Imber A. N. Santin J. M. Graham C. D. Putnam R. W. (2014). A HCO₃^−^-dependent mechanism involving soluble adenylyl cyclase for the activation of Ca^2+^ currents in locus coeruleus neurons. Biochim. Biophys. Acta 1842, 2569–2578. 10.1016/j.bbadis.2014.07.027 25092170 PMC4262627

[B32] Jacobs S. Ruusuvuori E. Sipilä S. T. Haapanen A. Damkier H. H. Kurth I. (2008). Mice with targeted Slc4a10 gene disruption have small brain ventricles and show reduced neuronal excitability. Proc. Natl. Acad. Sci. U.S.A. 105, 311–316. 10.1073/pnas.0705487105 18165320 PMC2224208

[B33] Lamprianou S. Vacaresse N. Suzuki Y. Meziane H. Buxbaum J. D. Schlessinger J. (2006). Receptor protein tyrosine phosphatase γ is a marker for pyramidal cells and sensory neurons in the nervous system and is not necessary for normal development. Mol. Cell. Biol. 26, 5106–5119. 10.1128/MCB.00101-06 16782895 PMC1489161

[B34] Lee S.-K. Occhipinti R. Moss F. J. Parker M. D. Grichtchenko I. I. Boron W. F. (2023). Distinguishing among HCO₃^−^, CO₃^=^, and H^+^ as substrates of proteins that appear to be “bicarbonate” transporters. J. Am. Soc. Nephrol. 34, 40–54. 10.1681/ASN.2022030289 36288904 PMC10103014

[B35] Lesage F. (2003). Pharmacology of neuronal background potassium channels. Neuropharmacology 44, 1–7. 10.1016/s0028-3908(02)00339-8 12559116

[B36] Lorenzetto E. Moratti E. Vezzalini M. Harroch S. Sorio C. Buffelli M. (2014). Distribution of different isoforms of receptor protein tyrosine phosphatase γ (Ptprg-RPTP γ) in adult mouse brain: upregulation during neuroinflammation. Brain Struct. Funct. 219, 875–890. 10.1007/s00429-013-0541-7 23536318

[B37] Michenkova M. Taki S. Blosser M. C. Hwang H. J. Kowatz T. Moss F. J. (2021). Carbon dioxide transport across membranes. Interface Focus 11, 20200090. 10.1098/rsfs.2020.0090 33633837 PMC7898146

[B38] Moss F. J. Wass A. B. Watterson S. J. Boron W. F. (2018). Sensing and transduction of acid-base disturbances by receptor protein tyrosine phosphatase γ. FASEB J. 32, 864–865. 10.1096/fasebj.2018.32.1_supplement.864.5

[B39] Müller S. Lamszus K. Nikolich K. Westphal M. (2004). Receptor protein tyrosine phosphatase ζ as a therapeutic target for glioblastoma therapy. Expert Opin. Ther. Targets 8, 211–220. 10.1517/14728222.8.3.211 15161428

[B40] Occhipinti R. Musa-Aziz R. Boron W. F. (2014). Evidence from mathematical modeling that carbonic anhydrase II and IV enhance CO₂ fluxes across *Xenopus* oocytes plasma membranes. Am. J. Physiol. Cell Physiol. 307, C841–C858. 10.1152/ajpcell.00049.2014 24965589 PMC4216938

[B41] Occhipinti R. Safaei S. Hunter P. Boron W. F. (2020). The Boron and De Weer model of intracellular pH regulation. Physiome. 10.36903/physiome.12871022

[B42] Parker J. C. (1983). Volume-responsive sodium movements in dog red blood cells. Am. J. Physiol. 244, C324–C330. 10.1152/ajpcell.1983.244.5.C324 6846523

[B43] Radu C. G. Nijagal A. McLaughlin J. Wang L. Witte O. N. (2005). Differential proton sensitivity of related G protein-coupled receptors T cell death-associated gene 8 and G2A expressed in immune cells. Proc. Natl. Acad. Sci. U. S. A. 102, 1632–1637. 10.1073/pnas.0409415102 15665078 PMC545089

[B44] Raimondo J. V. Irkle A. Wefelmeyer W. Newey S. E. Akerman C. J. (2012). Genetically encoded proton sensors reveal activity-dependent pH changes in neurons. Front. Mol. Neurosci. 5, 68. 10.3389/fnmol.2012.00068 22666186 PMC3364509

[B45] Rajendran V. M. Geibel J. Binder H. J. (1995). Chloride-dependent Na-H exchange. A novel mechanism of sodium transport in colonic crypts. J. Biol. Chem. 270, 11051–11054. 10.1074/jbc.270.19.11051 7744735

[B46] Rajendran V. M. Geibel J. Binder H. J. (1999). Role of Cl channels in Cl-dependent Na/H exchange. Am. J. Physiology - Gastrointest. Liver Physiology 276, G73–G78. 10.1152/ajpgi.1999.276.1.G73 9886981

[B47] Ransom B. R. (2000). Glial modulation of neural excitability mediated by extracellular pH: a hypothesis revisited. Prog. Brain Res. 125, 217–228. 10.1016/S0079-6123(00)25012-7 11098659

[B48] Ransom B. R. (2017). “The neuronal microenvironment,” in Medical physiology: a cellular and molecular approach. Editors Boron W. F. Boulpaep E. L. (Philadelphia, PA: Saunders Elsevier), 275–294.

[B49] Roos A. Boron W. F. (1981). Intracellular pH. Physiol. Rev. 61, 296–434. 10.1152/physrev.1981.61.2.296 7012859

[B50] Ruffin V. A. Taki S. Boron W. F. Moss F. J. (2025). Novel RPTPγ and RPTPζ splice variants from mixed neuron–astrocyte hippocampal cultures as well as from the hippocampi of newborn and adult mice. Front. Physiol. 15. 10.3389/fphys.2024.1406448 PMC11215419 38952869

[B51] Salameh A. I. Hübner C. A. Boron W. F. (2017). Role of Cl^−^-HCO₃^−^ exchanger AE3 in intracellular pH homeostasis in cultured murine hippocampal neurons, and in crosstalk to adjacent astrocytes. J. Physiol. (Lond.) 595, 93–124. 10.1113/JP272470 27353306 PMC5199729

[B52] Salameh A. I. Ruffin V. A. Boron W. F. (2014). Effects of metabolic acidosis on intracellular pH responses in multiple cell types. Am. J. Physiol. Regul. Integr. Comp. Physiol. 307, R1413–R1427. 10.1152/ajpregu.00154.2014 25209413 PMC4269672

[B53] Schneider J. W. Goetsch S. C. Leng X. Ludwig S. M. Russell J. L. Yang C.-P. (2012). Coupling hippocampal neurogenesis to brain pH through proneurogenic small molecules that regulate proton sensing G protein-coupled receptors. ACS Chem. Neurosci. 3, 557–568. 10.1021/cn300025a 22860225 PMC3400383

[B54] Schwiening C. J. Boron W. F. (1994). Regulation of intracellular pH in pyramidal neurones from the rat hippocampus by Na^+^-dependent Cl^−^-HCO₃^−^ exchange. J. Physiol. (Lond) 475, 59–67. 10.1113/jphysiol.1994.sp020049 8189393 PMC1160355

[B55] Siebens A. W. Boron W. F. (1989a). Depolarization-induced alkalinization in proximal tubules. I. Characteristics and dependence on Na^+^ . Am. J. Physiol. 256, F342–F353. 10.1152/ajprenal.1989.256.2.F342 2916666

[B56] Siebens A. W. Boron W. F. (1989b). Depolarization-induced alkalinization in proximal tubules. II. Effects of lactate and SITS. Am. J. Physiol. 256, F354–F365. 10.1152/ajprenal.1989.256.2.F354 2916667

[B57] Sinning A. Liebmann L. Kougioumtzes A. Westermann M. Bruehl C. Hübner C. A. (2011). Synaptic glutamate release is modulated by the Na^+^-driven Cl^−^/HCO_3_ ^−^ exchanger Slc4a8. J. Neurosci. 31, 7300–7311. 10.1523/JNEUROSCI.0269-11.2011 21593314 PMC6622604

[B58] Smith G. A. Brett C. L. Church J. (1998). Effects of noradrenaline on intracellular pH in acutely dissociated adult rat hippocampal CA1 neurones. J. Physiol. (Lond.) 512 (Pt 2), 487–505. 10.1111/j.1469-7793.1998.487be.x 9763638 PMC2231226

[B59] Somersalo E. Occhipinti R. Boron W. F. Calvetti D. (2012). A reaction-diffusion model of CO_2_ influx into an oocyte. J. Theor. Biol. 309, 185–203. 10.1016/j.jtbi.2012.06.016 22728674 PMC3471386

[B60] Taki S. (2024). Identification of novel murine RPTPγ and RPTPζ splice variants and the role of RPTPζ in modulating the neuronal and astrocytic intracellular pH response to metabolic or respiratory acidosis. Cleveland, OH: Case Western Reserve University Health Sciences. Available at: https://etd.ohiolink.edu/.

[B61] Taki S. Boron W. F. Moss F. J. (2024). Novel RPTPγ and RPTPζ splice variants from mixed neuron-astrocyte hippocampal cultures as well as from the hippocampi of newborn and adult mice. Front. Physiol. 15, 1406448. 10.3389/fphys.2024.1406448 38952869 PMC11215419

[B62] Thornell I. M. Michenkova M. Taki S. Bevensee M. O. Boron W. F. (2025). “Intracellular pH homeostasis,” in Seldin and giebisch’s the kidney: physiology and pathophysiology. Editors Alpern R. J. Caplan M. J. Moe O. W. Quaggin S. E. (London: Elsevier/Academic Press). Available at: https://www.barnesandnoble.com/w/seldin-and-giebischs-the-kidney-robert-j-alpern/1138252983 (Accessed August 29, 2023).

[B63] Xiong Z. Q. Saggau P. Stringer J. L. (2000). Activity-dependent intracellular acidification correlates with the duration of seizure activity. J. Neurosci. 20, 1290–1296. 10.1523/JNEUROSCI.20-04-01290.2000 10662818 PMC6772378

[B64] Yuen A. W. C. (2006). Low-grade chronic metabolic acidosis is a contributory mechanism in the development of chronic epilepsy. Epilepsy Behav. 8, 347–349. 10.1016/j.yebeh.2005.11.012 16459150

[B65] Zhao J. Hogan E. M. Bevensee M. O. Boron W. F. (1995). Out-of-equilibrium CO₂/HCO₃^−^solutions and their use in characterizing a new K^+^/HCO₃^−^ cotransporter. Nature 374, 636–639. 10.1038/374636a0 7715702

[B66] Zhao J. Zhou Y. Boron W. F. (2003). Effect of isolated removal of either basolateral HCO_3_ ^−^ or basolateral CO_2_ on HCO_3_ ^−^ reabsorption by rabbit S2 proximal tubule. Am. J. Physiol. Ren. Physiol. 285, F359–F369. 10.1152/ajprenal.00013.2003 12734099

[B67] Zhou Y. Skelton L. A. Xu L. Chandler M. P. Berthiaume J. M. Boron W. F. (2016). Role of receptor protein tyrosine phosphatase γ in sensing extracellular CO₂ and HCO₃^−^ . J. Am. Soc. Nephrol. 27, 2616–2621. 10.1681/ASN.2015040439 26839367 PMC5004642

[B68] Zhou Y. Zhao J. Bouyer P. Boron W. F. (2005). Evidence from renal proximal tubules that HCO₃^−^ and solute reabsorption are acutely regulated not by pH but by basolateral HCO₃^−^ and CO₂. Proc. Natl. Acad. Sci. U.S.A. 102, 3875–3880. 10.1073/pnas.0500423102 15728388 PMC553318

